# Cucurbitacins in Plant–Insect Interactions: Biosynthesis, Regulation, Ecological Functions, and Prospects for Crop Protection

**DOI:** 10.3390/plants15172660

**Published:** 2026-08-30

**Authors:** Qi Zhang, Yu-e Bai, Aoga Li

**Affiliations:** College of Forestry, Inner Mongolia Agricultural University, Hohhot 010018, China

**Keywords:** cucurbitacins, triterpenoid biosynthesis, molecular regulation, chemical defense, herbivore adaptation, agroecology, crop domestication, integrated pest management

## Abstract

Cucurbitacins are highly oxygenated tetracyclic triterpenoids characterized by intense bitterness, substantial structural diversity, and important consequences for plant–herbivore interactions. Although best known from Cucurbitaceae, cucurbitacins and related cucurbitane-type metabolites also occur in phylogenetically distant herbaceous and woody plants. Genetic and biochemical studies have validated several core biosynthetic steps, including cucurbitadienol formation by oxidosqualene cyclases and subsequent modification by cytochrome P450 monooxygenases, acyltransferases, and glycosyltransferases. Tissue-preferential basic helix–loop–helix transcription factors constitute the best-characterized regulatory layer, whereas the evidence supporting accessory regulators, transporters, and environmental responses varies from functional validation to transcriptomic or genomic prediction. From the plant perspective, cucurbitacins deter feeding or impair performance in many generalist and non-adapted herbivores. By contrast, their use as host-recognition cues and feeding stimulants by specialist diabroticite beetles reflects evolved herbivore adaptations involving perception, tolerance, metabolism, or sequestration rather than a second defensive function of the plant trait. Herbivore-induced cucurbitacin accumulation has been demonstrated in particular systems, although its regulatory mechanisms and ecological generality remain unresolved. Unlike previous reviews centered primarily on cucurbitacin chemistry, pharmacological activity, or individual biosynthetic pathways, this review integrates evidence-graded pathway reconstruction and molecular regulation with taxonomic distribution, insect adaptation, domestication, and agroecological consequences. Mechanistically, this review traces how scaffold formation, oxidative tailoring, conjugation, tissue-specific regulation, and transport give rise to contrasting ecological outcomes through herbivore-specific perception, tolerance, metabolism, and sequestration. We conclude that uniformly increasing or eliminating cucurbitacins is unlikely to provide broadly effective crop resistance because either direction may favor a different herbivore group. Future priorities include functional validation of candidate genes, spatially resolved metabolite analysis, comparative investigation of non-cucurbit lineages, and field evaluation involving generalist and specialist herbivores, crop quality, and non-target organisms. These advances will support context-specific fruit-quality improvement, behavioral pest control, and integrated pest management strategies rather than cucurbitacin manipulation as a stand-alone resistance approach.

## 1. Introduction

Plants produce a remarkable diversity of specialized metabolites that contribute to ecological adaptation, plant–environment interactions, and defense against herbivores and pathogens. Among these metabolites, terpenoids represent one of the largest and most structurally diverse groups of plant natural products. Triterpenoids derived from 2,3-oxidosqualene exhibit extensive structural diversity through alternative cyclization reactions followed by oxidation, acylation, glycosylation, and other modifications, resulting in compounds with distinct biological functions [1,2].

Cucurbitacins are highly oxygenated tetracyclic triterpenoids characterized by a cucurbitane-type carbon skeleton and intense bitterness. More than one hundred cucurbitacin-related compounds have been identified, including cucurbitacins A–T, glycosylated derivatives, and various oxidized, reduced, acylated, and rearranged forms [1,3]. Their biological effects depend not only on total concentration but also on compound identity, conjugation state, tissue localization, and accessibility to interacting organisms [4,5,6,7]. Cucurbitacin accumulation should therefore not be treated as a single uniform chemical or defensive trait.

Cucurbitacins are best known from the Cucurbitaceae, in which they have been reported in at least 100 species belonging to approximately 30 genera [8]. However, cucurbitacins and related cucurbitane-type triterpenoids also occur in several phylogenetically distant plant lineages, including *Iberis* (Brassicaceae), *Aquilaria* (Thymelaeaceae), and *Elaeocarpus* (Elaeocarpaceae) [8,9,10,11,12,13,14,15,16,17,18]. The identification of an independently evolved cucurbitacin pathway in *Iberis amara* demonstrated that similar defensive metabolites can arise through the recruitment and functional divergence of distinct oxidosqualene cyclases and cytochrome P450 enzymes [8]. This finding indicates that cucurbitacin biosynthesis did not necessarily originate from a single ancestral pathway but may have evolved convergently in different plant families. Nevertheless, molecular evidence remains concentrated in a few cucurbit crops, whereas reports from many non-Cucurbitaceae species are still based primarily on phytochemical identification or genomic prediction.

The molecular basis of cucurbitacin biosynthesis has been most extensively characterized in cucumber, melon, and watermelon. Studies in these species have identified scaffold-forming oxidosqualene cyclases, cytochrome P450 monooxygenases, acyltransferases, and tissue-preferential transcriptional regulators involved in cucurbitacin biosynthesis and bitterness control [19,20]. More recent work has revealed additional developmental, hormonal, and stress-responsive regulators of cucurbitacin accumulation [21,22]. However, the strength of evidence differs substantially among pathway components. Some enzymes and regulators have been supported by genetic and biochemical validation, whereas others remain candidates inferred from genomic location, sequence homology, co-expression, or metabolite association [19,20,21,22,23,24,25,26]. Distinguishing experimentally validated components from predicted candidates is therefore essential for interpreting the current biosynthetic and regulatory framework.

From the perspective of the plant, cucurbitacins primarily function as defensive metabolites because they deter feeding or reduce the performance of many generalist and non-adapted herbivores. Cucurbitacin C accumulation in cucumber leaves has been associated with resistance to the two-spotted spider mite, *Tetranychus urticae* [27]. Cucurbitacin B reduces survival, longevity, fecundity, and population growth of the melon aphid, *Aphis gossypii*, and affects detoxification-related responses [28,29]. Cucurbitacin E isolated from *Citrullus colocynthis* also causes feeding inhibition, mortality, and developmental and reproductive impairment in *Spodoptera litura* [30]. Nevertheless, evidence obtained using purified compounds, crude extracts, detached tissues, or artificial diets cannot always be extrapolated directly to intact plants, in which compound localization, glycosylation, interactions with other metabolites, and herbivore behavior may alter the biological outcome [5,6,27,30,31,32,33].

Specialist diabroticite beetles present a contrasting case. Several of these beetles have evolved sensory and physiological adaptations that allow them to use cucurbitacins as host-location cues and feeding stimulants and, in some cases, to absorb, transform, or sequester the compounds after ingestion [34,35,36]. These responses should not be interpreted as a second adaptive function of cucurbitacins for the plant. Instead, they represent herbivore counteradaptations to a plant defensive trait. This distinction is agronomically important because diabroticite beetles include major crop pests. For example, yield losses and management costs associated with the western corn rootworm, *Diabrotica virgifera virgifera*, have been estimated to exceed USD 1 billion annually [37]. Understanding cucurbitacin-mediated interactions therefore requires consideration of both plant defense and the evolutionary adaptations of the herbivores that exploit these metabolites.

The opposing responses of generalist and specialist herbivores create an important constraint on cucurbitacin-based crop improvement. Constitutively increasing cucurbitacin concentrations may strengthen resistance to some generalists while increasing host location or feeding by adapted specialists. Conversely, reducing cucurbitacin accumulation to improve fruit palatability or decrease specialist attraction may increase susceptibility to non-adapted herbivores. Thus, neither increasing nor decreasing total cucurbitacin content is likely to provide universally effective crop protection. The outcome will depend on the local herbivore community, the relative abundance of specialist and generalist species, plant tissue and developmental stage, compound composition, and interactions with other defensive traits. Cucurbitacin-based interventions should therefore be evaluated within an agroecological and integrated pest management framework rather than as isolated, single-trait solutions.

Current knowledge also remains uneven across plant lineages, experimental systems, and levels of evidence. Functional studies are concentrated mainly in cultivated Cucurbitaceae, whereas the evolutionary origins, ecological functions, and regulatory mechanisms of cucurbitacins in non-cucurbit and woody species remain comparatively poorly characterized [8,13,19,20,21,22]. In addition, relatively few studies have simultaneously quantified cucurbitacin concentration, herbivore behavior, herbivore performance, plant fitness, and effects on non-target organisms under field conditions [31,32,33]. These limitations restrict the translation of pathway knowledge into reliable crop-protection strategies.

Previous reviews have mainly focused on cucurbitacin chemistry, pharmacological activities, or individual biosynthetic pathways [1,3]. In contrast, this review integrates structural diversity, taxonomic distribution, evolutionary origins, biosynthesis, molecular regulation, plant defense, specialist-herbivore adaptation, domestication, and agroecological constraints. Particular attention is given to distinguishing experimentally validated findings from genomic or transcriptomic candidates and to evaluating how evidence obtained from different experimental systems supports ecological and agronomic conclusions. By integrating phytochemistry, plant molecular biology, evolutionary ecology, and crop protection, this review aims to define both the potential and the limitations of cucurbitacin-centered strategies for sustainable pest management.

### Literature Search Strategy

The relevant literature was searched in the Web of Science Core Collection, Scopus, PubMed, and Google Scholar for studies published up to July 2026. The principal Boolean search framework was (“cucurbitacin*” OR “cucurbitane-type triterpenoid*”) AND (“biosynth*” OR “regulat*” OR “transport*” OR “distribution” OR “evolution” OR “plant–insect interaction*” OR “plant–herbivore interaction*” OR “herbivor*” OR “adaptation” OR “domestication” OR “breeding” OR “crop protection”). For insect-focused searches, this framework was supplemented with (“insect*” OR “beetle*” OR “aphid*” OR “mite*” OR “lepidopter*” OR “Diabrotica” OR “Acalymma” OR “Epilachna” OR “Tetranychus” OR “Aphis” OR “Spodoptera”). Search syntax was adjusted where necessary to meet the requirements of each database. The operator NOT was not used because it could exclude interdisciplinary studies relevant to plant chemistry, biosynthesis, ecology, or crop protection. Additional publications were identified by screening the reference lists of relevant original articles and reviews.

Titles and abstracts were initially screened for relevance, followed by full-text assessment. Peer-reviewed studies were included when they provided evidence concerning cucurbitacin identification, distribution, biosynthesis, molecular regulation, transport, plant–insect interactions, domestication, breeding, or crop protection. Studies focused exclusively on pharmaceutical or clinical activities were excluded unless they provided information directly relevant to compound identification, biosynthesis, or plant ecological function. Duplicate reports and publications lacking sufficient information on plant material, compound identity, experimental system, or biological response were excluded from the evidence synthesis. Evidence tiers were assigned relative to the research question specified for each study and were classified as Validated, Supported, Candidate, or Contextual according to the criteria defined in Supplementary Table S1. Because of the broad interdisciplinary scope and heterogeneity of the available evidence, the literature was synthesized as a structured narrative review rather than a formal meta-analysis. Within this framework, the review is organized along a mechanistic continuum connecting cucurbitacin biosynthetic diversification and tissue-level deployment in plants with perception, metabolic processing, and sequestration in herbivores. A study-level evidence matrix summarizing the research question, experimental system, main finding, evidence tier, and principal limitation of each included study is provided in Supplementary Table S1.

## 2. Chemical Diversity and Distribution of Cucurbitacins

Cucurbitacins comprise a chemically diverse group of highly oxygenated tetracyclic triterpenoids that exhibit intense bitterness and a broad range of biological and ecological activities [1,3]. Although they are most frequently associated with Cucurbitaceae, cucurbitacins and related cucurbitane-type metabolites occur in multiple plant families [8,9,10,11,12,13,14,15,16,17,18]. Their occurrence is taxonomically discontinuous, and their qualitative composition and quantitative abundance vary among species, genotypes, organs, developmental stages, and environmental conditions [4,6,8,23,38,39,40,41,42,43]. Cucurbitacin profiles should therefore be regarded as chemically heterogeneous and spatially structured components of plant specialized metabolism rather than as uniform chemotaxonomic or defensive traits. This heterogeneity is important for plant–herbivore interactions because ecological outcomes depend on compound identity, conjugation state, tissue accessibility, and herbivore sensory and metabolic adaptations, rather than on total cucurbitacin concentration alone.

### 2.1. Structural Diversity

**Figure 1:**
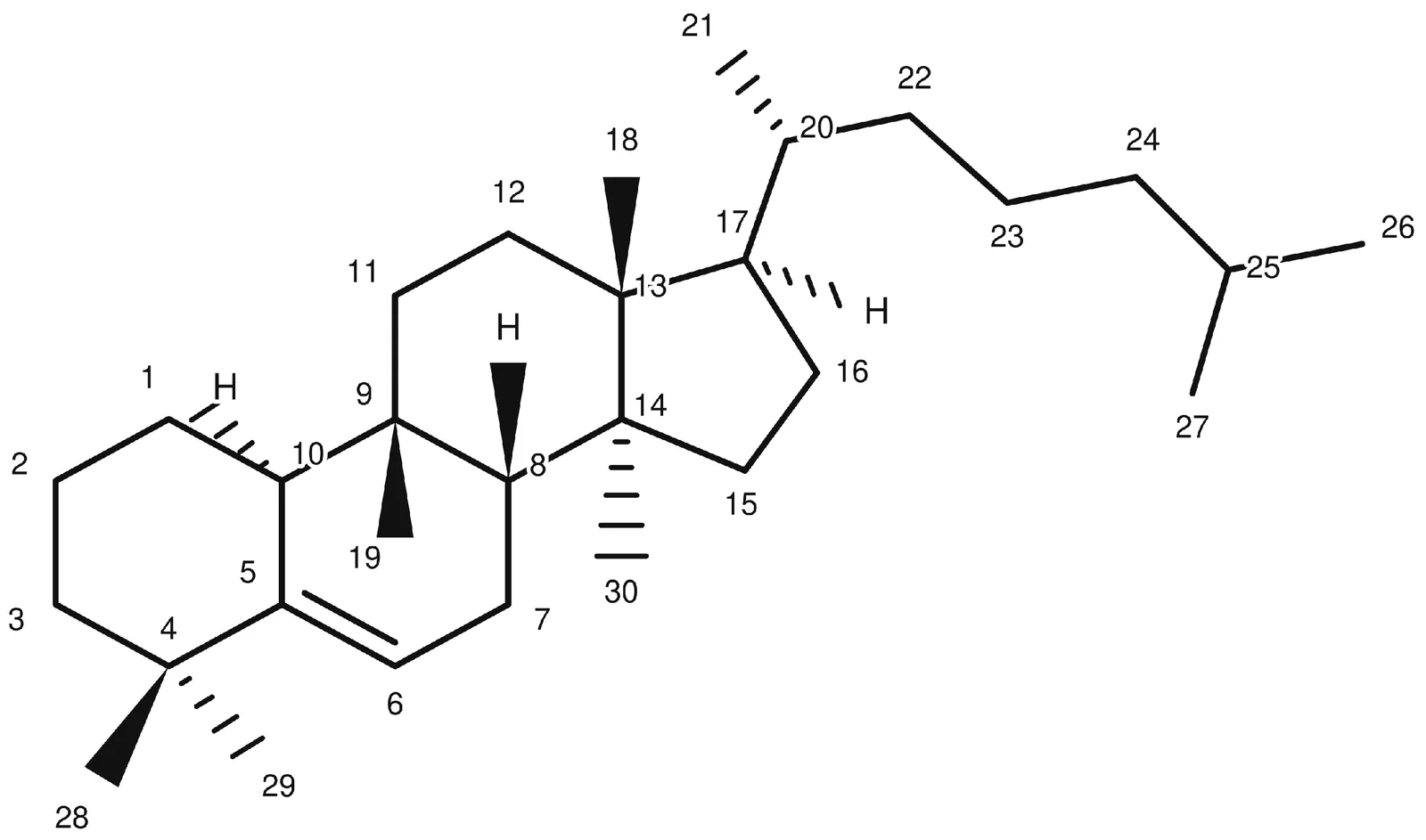
Cucurbitane [19(10→9β)-abeo-10α-lanost-5-ene] skeleton with the standard carbon-numbering system. The numbers 1–30 indicate the standard carbon positions of the cucurbitane skeleton. Adapted from Benka et al. [47] under the terms of the CC BY 4.0 license.

Cucurbitacins are characterized by a cucurbitane-type tetracyclic carbon skeleton, generally derived from cucurbitadienol, and are diversified by multiple downstream reactions [1,25,44,45,46]. Oxidation, hydroxylation, acylation, glycosylation, side-chain reduction, and structural rearrangement generate a large number of aglycones, glycosides, and partially modified intermediates. Cucurbitacins are conventionally designated by letters from A to T, although this nomenclature does not encompass the growing number of naturally occurring analogues. The cucurbitane core and its standard carbon-numbering system are shown in Figure 1 to facilitate interpretation of the position-specific structural modifications discussed below.

Modern metabolite profiling indicates that cucurbitacin composition is considerably more complex than the small number of major letter-designated compounds would suggest, although the confidence of individual annotations varies. Chen et al. developed a rapid screening method to characterize the distribution of cucurbitacins and their glycosides in cucumber leaves, roots, stems, and flowers. The method combined high-performance liquid chromatography/quadrupole-time-of-flight mass spectrometry (HPLC-Q-TOF-MS) with in-source fragmentation (ISF) and alkali-adduct formation. Using diagnostic fragmentation patterns and comparisons with reference spectra, the study screened 40 putative cucurbitacins and cucurbitacin glycosides and proposed an organ-specific metabolic distribution network [4]. The significance of this study lies not in the number of detected features alone, but in showing that cucumber contains a broader and more spatially structured cucurbitacin profile than is represented by its major named compounds. Complementary isolation and structural characterization of ten cucurbitacin C analogues from bitter cucumber leaves, including seven previously undescribed compounds, provided stronger structural evidence for part of this chemical diversity [48].

Glycosylation and side-chain modification provide additional sources of diversity. The cucumber UDP-glycosyltransferase UGT73AM3 catalyzes developmentally regulated glucosylation of a bitter cucurbitacin intermediate, linking fruit development with the conversion and storage of bitter triterpenoids [5]. Side-chain saturation, isomerization, and variation in oxidation patterns have also been demonstrated in *Citrullus naudinianus*, *Picrorhiza kurrooa*, and *Cucurbita texana* [47,49,50]. These modifications may alter solubility, intracellular transport, stability, tissue storage, and recognition or detoxification by herbivores. Accordingly, studies of biological activity should report compound identity, analytical confidence, purity, dose, application method, and conjugation state. The generic term “cucurbitacin treatment” is insufficient when structurally distinct compounds may elicit different behavioral or physiological responses.

### 2.2. Taxonomic Distribution and Evolutionary Origins

Cucurbitacins have been reported in at least 100 species belonging to approximately 30 genera of Cucurbitaceae [8]. Cultivated cucurbits containing cucurbitacins or related pathway components include cucumber (*Cucumis sativus*), melon (*Cucumis melo*), watermelon (*Citrullus lanatus*), squash and pumpkin (*Cucurbita* spp.), bottle gourd (*Lagenaria siceraria*), and luffa (*Luffa* spp.). Reports also extend to wild, bitter, or medicinal taxa, including *Citrullus colocynthis*, *Cucurbita texana*, *Momordica*, *Trichosanthes*, *Hemsleya* (Cucurbitaceae), *Ecballium*, and *Bryonia*. This diversity encompasses domesticated crops selected for reduced fruit bitterness as well as wild and medicinal plants in which high cucurbitacin accumulation has been retained.

Distribution within Cucurbitaceae is nevertheless neither uniform nor predictable solely from taxonomic position. Comparative liquid chromatography–mass spectrometry profiling of ten species belonging to six cucurbitaceous genera detected distinct species-specific combinations of cucurbitacins B, E, I, and E-glucoside [38]. Similarly, analysis of 69 cucumber inbred lines detected substantial genotypic variation in cucurbitacins A–E and I, with cucurbitacin C present in only six lines [39]. These results show that closely related species or genotypes may differ markedly in their dominant compounds, whereas similar cucurbitacins can occur in more distantly related taxa.

**Figure 2:**
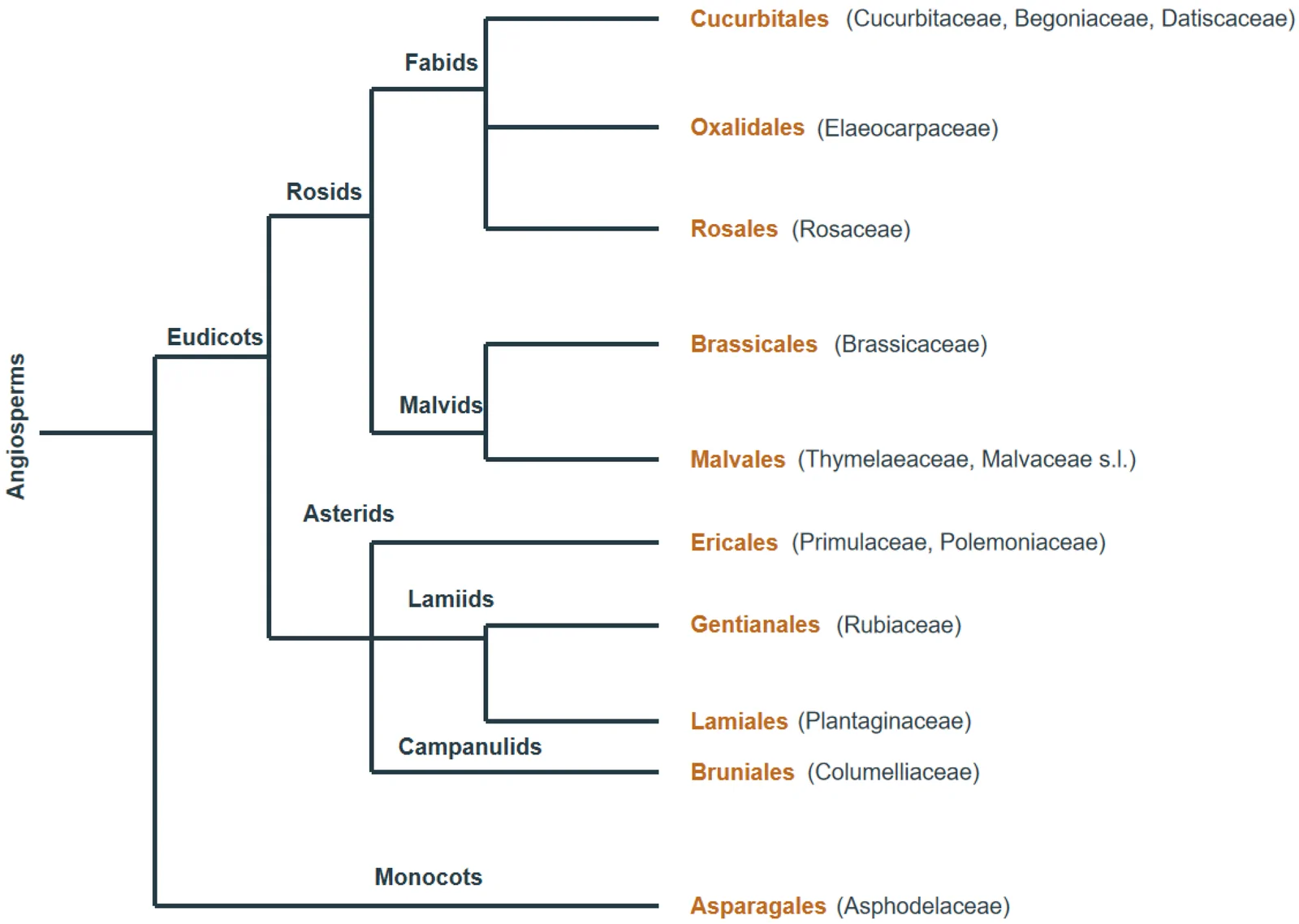
Phylogenetic distribution of cucurbitacin-containing angiosperm families. The simplified topology was independently redrawn based on Dong et al. [8]. Orders containing families in which cucurbitacins or related cucurbitane-type metabolites have been reported are highlighted in orange, with the corresponding families shown in parentheses. Branch lengths are schematic and are not proportional to evolutionary time.

Outside Cucurbitaceae, cucurbitacins and related cucurbitane-type metabolites have been reported from at least 15 taxonomically distant plant families (Figure 2). Representative examples include *Iberis* in Brassicaceae, *Begonia* in Begoniaceae, Elaeocarpus in *Elaeocarpaceae*, and *Aquilaria* in Thymelaeaceae, as well as members of Rosaceae, Rubiaceae, and other distantly related angiosperm lineages. Their fragmented distribution across the angiosperm phylogeny indicates a discontinuous taxonomic occurrence and suggests multiple evolutionary origins of cucurbitacin biosynthesis. In *Iberis amara*, phylogenetic analysis and functional characterization of oxidosqualene cyclases and cytochrome P450 enzymes demonstrated that cucurbitacin biosynthesis evolved independently from the pathway found in Cucurbitaceae [8]. The Brassicaceae and Cucurbitaceae pathways recruit different enzyme lineages and follow different early oxidation routes, supporting convergent evolution rather than uninterrupted inheritance of a single ancestral cucurbitacin pathway [8].

Taxonomic surveys must distinguish among chemically confirmed cucurbitacins, putatively annotated metabolites, and broadly defined cucurbitane-type triterpenoids. Identification supported by authentic standards, diagnostic tandem mass spectrometry, nuclear magnetic resonance, or biochemical reconstruction provides stronger evidence than annotation based only on accurate mass, database matching, or transcriptomic association. This distinction is particularly important in poorly studied families, where structurally related triterpenoids may otherwise be assigned incorrectly to established cucurbitacin classes.

### 2.3. Organ-, Ontogenetic-, and Genotype-Specific Distribution

Most available evidence for organ- and ontogenetic variation currently comes from Cucurbitaceae, particularly cucumber [4,5,6,19,40,41]. Cucurbitacins are heterogeneously distributed among roots, stems, leaves, flowers, fruits, seeds, and vascular tissues [4,6,40,41]. Wild and bitter genotypes often accumulate them across several organs, whereas domesticated cultivars commonly exhibit reduced fruit bitterness while retaining some cucurbitacin production in roots or vegetative tissues [19,32,33]. Such spatial separation is ecologically and agronomically important because it can preserve defense in vulnerable organs while limiting bitterness in edible tissues [19,32,33].

Changes associated with organ formation, tissue maturation, or plant age represent ontogenetic trajectories rather than environmentally inducible plasticity [4,40,41,51]. These developmentally programmed changes should be distinguished from reversible or stimulus-dependent responses to drought, hormones, or herbivore feeding, which are discussed in Section 2.4.

Quantitative studies in cucumber illustrate distinct organ- and age-dependent trajectories. Cucurbitacin C and 23,24-dihydrocucurbitacin C were detected in leaves and stems, whereas cucurbitacin C concentrations decreased progressively as leaves aged [40]. Broader HPLC-Q-TOF-MS screening also revealed different distributions of cucurbitacins and their glycosides among cucumber leaves, roots, stems, and flowers [4]. These results indicate that individual cucurbitacins follow compound- and tissue-specific ontogenetic patterns rather than simply undergoing generalized “developmentally dynamic” changes.

Strong heterogeneity also occurs within cucumber fruit. In the bitter cultivar ‘Hanzil’, cucurbitacin C concentration increased with fruit enlargement and differed markedly among pericarp tissues. The endocarp contained the highest concentration, followed by the mesocarp and exocarp, whereas cucurbitacin C and its glycoside were undetectable in the non-bitter cultivar ‘Vlaspik’ [41]. This complex tissue- and developmental-stage-dependent pattern in cucumber contrasts with the classical case described in Cucurbita, in which fruit bitterness was reported as a simple Mendelian trait controlled by a single locus with two alleles, with bitterness behaving as the recessive phenotype [42]. The contrast indicates that the genetic architecture of bitterness cannot be generalized across cucurbit crops.

Genotype-dependent variation has been documented in cucumber and luffa. Among 69 cucumber inbred lines, cucurbitacin C was restricted to a small subset of genotypes, whereas volatile and morphological profiles showed additional independent patterns [39]. In *Luffa acutangula*, integrated metabolomic and transcriptomic analyses comparing bitter and non-bitter fruits detected 131 differentially accumulated metabolites. Isocucurbitacin B, cucurbitacin D, 23,24-dihydrocucurbitacin E, and cucurbitacin F were markedly more abundant in the bitter genotype, together with increased expression of *Bi*, cytochrome P450, and acyltransferase genes [23]. These results demonstrate that fruit bitterness may reflect coordinated changes in several cucurbitacins rather than variation in a single dominant compound.

Subcellular compartmentation provides an additional level of spatial organization. The cucumber multidrug and toxic compound extrusion/detoxification transporter CsMATE1 is physically associated and transcriptionally co-regulated with cucurbitacin C biosynthetic genes and mediates vacuolar sequestration of cucurbitacin C in leaf mesophyll cells [6]. Coordination between biosynthesis and transport may reduce autotoxicity while maintaining a stored chemical pool that becomes accessible after cellular disruption. Ecological exposure therefore depends on subcellular storage and release as well as on bulk tissue concentration.

### 2.4. Environmental and Herbivore-Induced Plasticity

Environmental plasticity refers here to changes in cucurbitacin concentration or composition produced in response to external stimuli, rather than unidirectional changes associated with normal organ development. Temperature, water status, mineral nutrition, plant-growth regulators, hormonal signals, and herbivory have all been associated with bitterness or cucurbitacin-related responses [6,21,23,31,43,51,52,53,54,55,56], but the different forms of evidence do not support equivalent conclusions. Direct quantification of an individual cucurbitacin before and after a controlled stimulus provides evidence of metabolite induction or suppression. By contrast, changes in bitterness, pathway-gene expression, or transcription-factor activity alone indicate a possible regulatory response but do not demonstrate a corresponding change in cucurbitacin concentration [6,23,31,43,52,53]. The examples below are evaluated according to these distinctions.

The most convincing mechanistic evidence for environmentally responsive transcriptional regulation currently comes from *Luffa acutangula*. Drought and abscisic acid (ABA) treatments increased the expression of *Bi*, cytochrome P450, and acyltransferase genes, while yeast one-hybrid and dual-luciferase assays demonstrated that the ABA-responsive transcription factor LaAREB1 binds to and activates the *LaBi* promoter [23]. These experiments establish direct regulation of a core biosynthetic gene by ABA signaling. However, because transcriptional activation and metabolite accumulation are not equivalent measurements, targeted quantification of individual cucurbitacins is still required to determine the resulting chemical response. Associations between CsMATE1 expression and ABA- or drought-related conditions similarly suggest environmentally coordinated transport but do not alone establish increased cucurbitacin C accumulation [6].

For herbivory, an increase in cucurbitacin concentration in a tissue following herbivore feeding is sufficient to demonstrate herbivore-induced accumulation. Additional evidence connecting perception, signaling, gene regulation, insect performance, and plant fitness is necessary only when claiming a specific regulatory mechanism or an adaptive defensive function. In bitter cucumber, Agrawal et al. found that feeding by the two-spotted spider mite, *Tetranychus urticae*, increased cucurbitacin C in locally damaged cotyledons and in an undamaged systemic leaf, providing direct evidence of local and systemic induction [43]. In zucchini, leaf damage increased cucurbitacin B and D concentrations, providing additional evidence of damage-induced accumulation [52]. However, induction is not universal across plant–herbivore combinations. McCloud et al. detected no cucurbitacin accumulation inside or outside the feeding trenches produced by the squash beetle, *Epilachna borealis* [53]. Likewise, feeding by *Diabrotica balteata* larvae caused only minor changes in cucurbitacin concentrations in the cucumber varieties and tissues examined [31].

These apparently contrasting findings indicate that herbivore-induced cucurbitacin accumulation depends on plant species and genotype, herbivore identity, sampled organ, local versus systemic tissue, and the timing of measurement. They do not justify describing cucurbitacins as either universally inducible or universally constitutive defenses. Instead, studies should distinguish clearly among direct metabolite induction, transcriptional responses without metabolite confirmation, and ecological effects on herbivore behavior or performance. The ecological consequences of this context dependence, particularly the contrasting responses of generalist and specialist herbivores, are examined in Section 5.

## 3. Biosynthetic Pathways of Cucurbitacins

**Figure 3:**
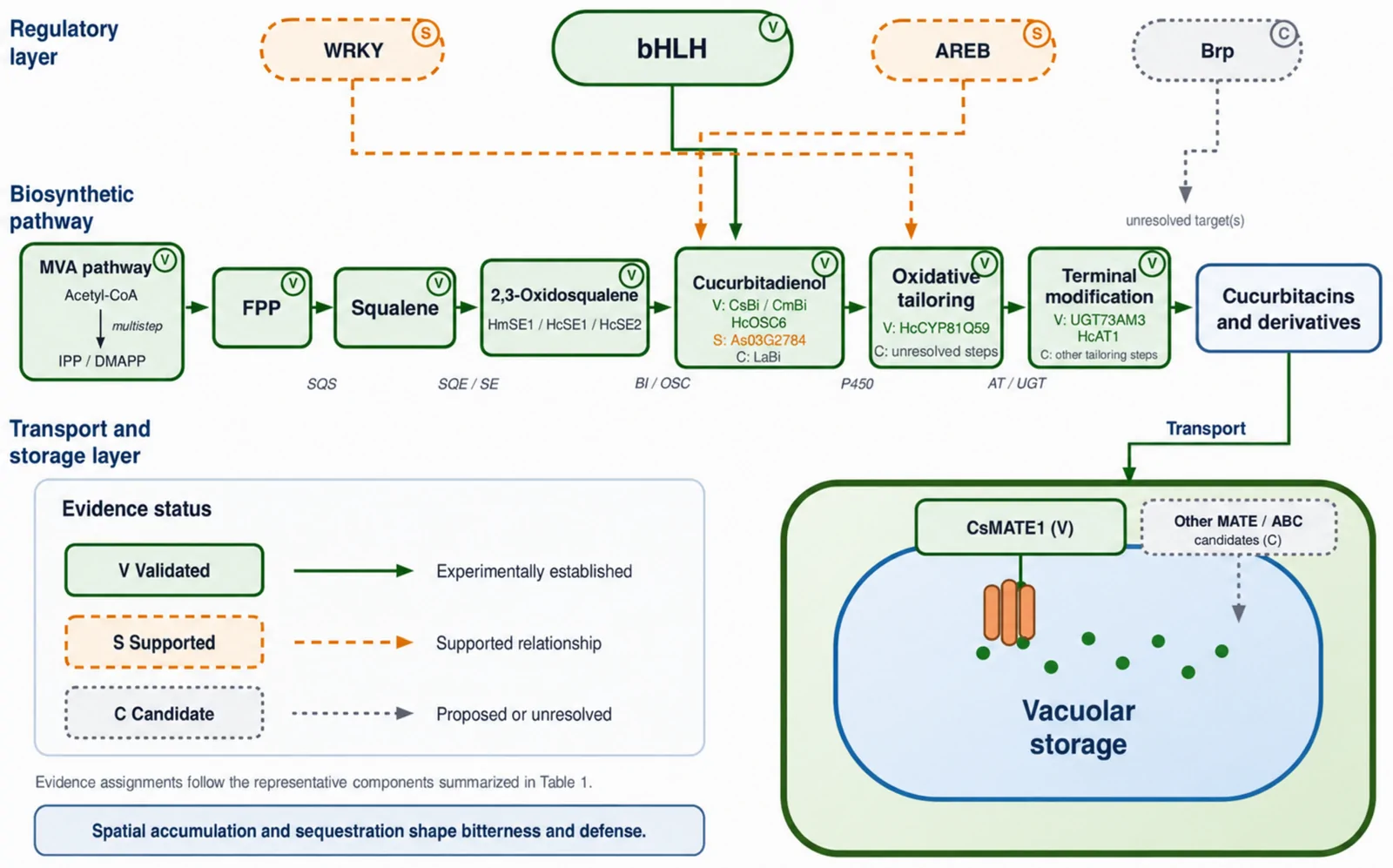
Overview of cucurbitacin biosynthesis and molecular regulation. The cytosolic mevalonate pathway supplies precursors for the formation of farnesyl diphosphate, squalene, and 2,3-oxidosqualene. Within the MVA pathway box, the downward arrow indicates the multistep conversion of acetyl-CoA to IPP and DMAPP rather than a decrease in their abundance. A Bi-type oxidosqualene cyclase catalyzes cucurbitadienol formation, followed by cytochrome P450-mediated oxidation and terminal modification by acyltransferases, UDP-glycosyltransferases, and related enzymes. Tissue-preferential bHLH transcription factors represent the best-characterized regulators, whereas WRKY and AREB factors provide additional regulatory inputs. Cucurbitacins and their derivatives may subsequently undergo intracellular transport and vacuolar sequestration; MATE-mediated transport in cucumber is shown here as a representative characterized example. Abbreviations: MVA, mevalonate; IPP, isopentenyl diphosphate; DMAPP, dimethylallyl diphosphate; FPP, farnesyl diphosphate; SQS, squalene synthase; SQE/SE, squalene epoxidase; OSC, oxidosqualene cyclase; AT, acyltransferase; UGT, UDP-glycosyltransferase; MATE, multidrug and toxic compound extrusion; ABC, ATP-binding cassette.

Cucurbitacin biosynthesis represents a specialized branch of plant triterpenoid metabolism [1,2]. The pathway can be divided into four major modules: precursor formation through the cytosolic mevalonate pathway, cyclization of 2,3-oxidosqualene into cucurbitadienol, oxidative modification by cytochrome P450 monooxygenases, and terminal acylation, glycosylation, reduction, or transport [8,19,20,24,25,44,45,46,57,58,59]. The upstream precursor-producing reactions are broadly conserved, whereas downstream enzymes differ substantially among plant lineages [2,8,20,25,46]. Consequently, cucurbitacin diversity is generated through lineage- and tissue-specific combinations of oxidosqualene cyclases, P450s, transferases, oxidoreductases, and transport-associated proteins [6,8,19,20,25,46,59]. To distinguish functional confirmation from prediction, components are classified as validated, supported, or candidate according to the criteria defined in Section 3.5 and Table 1. An overview of the major biosynthetic steps, transcriptional regulators, and transport processes associated with cucurbitacin accumulation is presented in Figure 3.

### 3.1. Precursor Formation Through the Mevalonate Pathway

The cucurbitacin carbon skeleton originates primarily from the cytosolic mevalonate pathway [2,24,25,44]. Acetyl coenzyme A (acetyl-CoA) is converted sequentially into 3-hydroxy-3-methylglutaryl coenzyme A (HMG-CoA), mevalonate, isopentenyl diphosphate, dimethylallyl diphosphate, and farnesyl diphosphate. Two farnesyl diphosphate molecules are then condensed by squalene synthase to form squalene, which is oxidized by squalene epoxidase to produce 2,3-oxidosqualene.

Because 2,3-oxidosqualene is also required for phytosterol and other triterpenoid pathways, cucurbitacin biosynthesis competes with essential sterol metabolism for a shared precursor pool. Comparative transcriptomic and biochemical analyses in *Hemsleya macrosperma* confirmed the activity of the squalene epoxidase HmSE1 and linked upstream pathway-gene expression with cucurbitacin IIa accumulation [24]. Functional characterization of HcSE1 and HcSE2, together with modular enhancement of the mevalonate pathway, further increased 2,3-oxidosqualene availability and cucurbitacin IIa intermediate production in engineered yeast [25].

Related heterologous yeast reconstructions showed that increasing precursor supply, optimizing cucurbitadienol synthase expression, and reducing competition from the sterol branch can raise the production of cucurbitane-type intermediates [60,61]. These studies clarify biochemical constraints on pathway flux; however, because they were conducted in microbial production systems—and some targeted mogrol rather than cucurbitacins—they do not demonstrate that increasing cucurbitacin biosynthesis would improve crop resistance or agronomic performance.

### 3.2. Formation of the Cucurbitane Skeleton

The first committed step is the cyclization of 2,3-oxidosqualene into cucurbitadienol by a specialized oxidosqualene cyclase, commonly designated Bi, CBS, CPQ, or a species-specific OSC. In pumpkin, CPQ was shown to produce cucurbitadienol, whereas the related CPX enzyme produced cycloartenol for phytosterol biosynthesis [44]. This established that cucurbitacin formation is initiated by a specialized cyclase distinct from sterol-associated OSCs.

Functional studies in *Siraitia grosvenorii*, bitter gourd, and *Trichosanthes cucumerina* subsequently confirmed that closely related OSCs can produce different cucurbitane, multiflorane, oleanane, or sterol scaffolds [45,46,57]. Therefore, phylogenetic position or gene annotation alone is insufficient to assign OSC function, and heterologous product identification remains necessary.

HcOSC6 from *Hemsleya chinensis* is among the best-characterized cucurbitadienol synthases. Site-directed mutagenesis identified E246, M261, and D490 as important residues affecting catalytic activity [58]. In the native plant, *HcOSC6* overexpression increased cucurbitadienol accumulation, whereas CRISPR/Cas9 editing reduced its abundance [62]. These results provide direct genetic evidence for its role in cucurbitacin biosynthesis.

### 3.3. Oxidative Tailoring by Cytochrome P450 Monooxygenases

Cucurbitadienol lacks the extensive oxygenation characteristic of mature cucurbitacins. Cytochrome P450 monooxygenases introduce hydroxyl, carbonyl, and other oxygen-containing groups into the tetracyclic nucleus and side chain, thereby generating much of the structural diversity within the pathway [8,25,46].

In bitter gourd, CYP81AQ19 catalyzes C-23 hydroxylation, whereas CYP88L7 and CYP88L8 catalyze different combinations of C-7 and C-19 hydroxylation reactions [46]. These enzymes demonstrate how differences in P450 regioselectivity can generate distinct cucurbitane structures.

In *H. chinensis*, HcCYP87D20 and HcCYP81Q59 were functionally characterized in engineered yeast and *Nicotiana benthamiana*. Their combined expression with HcSEs and HcOSC6 enabled the production of oxygenated cucurbitacin IIa intermediates and clarified several pathway reactions [25].

P450 activity can represent an important constraint in heterologous pathway reconstruction because these enzymes depend on appropriate reductases, membrane localization, oxygen supply, and cellular redox conditions [25,46]. Candidate P450s identified through co-expression or genomic proximity should therefore be regarded as provisional until their functions are confirmed by heterologous expression, substrate-feeding assays, product identification, and preferably genetic validation. Accordingly, genes supported only by co-expression or genomic proximity are classified as candidates in Table 1.

### 3.4. Acylation, Glycosylation, and Pathway Branching

Following P450-mediated oxidation, acyltransferases, glycosyltransferases, oxidoreductases, and related enzymes generate additional cucurbitacin derivatives. These terminal modifications influence solubility, stability, intracellular localization, transport, storage, and biological activity.

In *H. chinensis*, HcAT1 was shown to catalyze an acetylation step associated with cucurbitacin IIa biosynthesis [25]. In *Cucurbita pepo*, hairy-root cultures—genetically transformed root cultures induced by *Rhizobium rhizogenes*, rather than the root-hair zone—were used as a controlled system to test selected P450s and acyltransferases. Expression of these enzymes redirected endogenous intermediates and altered the relative abundance of cucurbitacins B, D, E, and I [59].

Because *R. rhizogenes* transformation can itself alter host metabolism, hairy-root results require controls that separate enzyme-specific effects from transformation effects. In *C. pepo*, transformation caused extensive transcriptomic and metabolomic changes and reduced endogenous cucurbitacin accumulation in transformed roots [63]. Therefore, both untransformed roots and empty-vector hairy-root lines are required for interpreting pathway-reconstruction experiments.

Glycosylation provides another major source of diversity. Studies of related cucurbitane glycosides in *S. grosvenorii* demonstrate that sequential glycosylation can generate highly modified products [45]. However, enzymes involved in mogroside biosynthesis should not automatically be assigned equivalent roles in cucurbitacin pathways. Most cucurbitacin glycosides and acylated derivatives still lack experimentally validated biosynthetic enzymes.

### 3.5. Genomic Organization, Evolution, and Evidence Assessment

Cucurbitacin biosynthetic genes are frequently located in partially clustered genomic regions containing a Bi-type OSC, P450s, acyltransferases, oxidoreductases, or transport-related genes [8,19,20,26,59]. However, not every gene within these regions necessarily participates in cucurbitacin production, and several essential genes may occur elsewhere in the genome [8,24,25,59].

Pathway organization varies among plant species. In *C. pepo*, major pathway genes occur in two chromosomal regions [59], whereas transcriptomic studies in *Hemsleya* species identified numerous dispersed candidates, only some of which have been functionally validated [24,25]. These patterns suggest that cucurbitacin pathways have evolved through gene duplication, loss, translocation, recruitment, and functional divergence.

Strong pathway assignment requires complementary evidence. Genomic location, phylogenetic placement, co-expression, and transcript–metabolite correlations are useful for candidate discovery but do not demonstrate enzymatic or regulatory function. In this review, direct product identification following heterologous expression, purified-enzyme assays, genetic gain- or loss-of-function analyses, or transport assays is treated as functional validation; promoter binding, transient activation, and pathway reconstruction without native genetic confirmation are treated as supporting evidence.

Reconstruction studies show that pathway modules can be combined under controlled conditions [25,59,60,61], but technical feasibility is not an agronomic rationale. Because higher cucurbitacin levels may deter generalists while stimulating adapted specialists, pathway manipulation should be considered only after ecological trade-offs, edible-tissue quality, non-target effects, and field context are evaluated (Section 5 and Section 6). Accordingly, engineering strategies are not detailed in this biosynthesis section.

Overall, the pathway comprises a conserved precursor module, a pathway-committing cucurbitadienol synthase, P450-mediated oxidation, and lineage-specific terminal modifications. Many branch points, transferases, glycosyltransferases, and transport processes remain unvalidated. Linking individual structures to biological effects is essential, but changing pathway flux cannot be assumed to improve crop protection.

Table 1 summarizes gene-specific evidence; validation in one taxon is not automatically generalized to homologues in another lineage.

**Table 1:** Experimentally validated, supported, and candidate genes associated with cucurbitacin biosynthesis, modification, transport, and regulation in representative plant species.

Species	Gene	Protein or Gene Class	Assigned Function	Evidence Level	Key Evidence	Reference
*Cucumis sativus*	*CsBi*	Oxidosqualene cyclase	Catalyzes cucurbitadienol formation and commits 2,3-oxidosqualene to cucurbitacin C biosynthesis	Validated	Heterologous expression, metabolite analysis, genetic association	[19]
*C. sativus*	*CsBl*	bHLH transcription factor	Predominantly regulates cucurbitacin C biosynthesis in leaves	Validated	Genetic analysis, promoter binding, transcriptional activation	[19]
*C. sativus*	*CsBt*	bHLH transcription factor	Predominantly regulates fruit bitterness and cucurbitacin C pathway-gene expression	Validated	Genetic analysis, promoter binding, phenotype association	[19]
*C. sativus*	*UGT73AM3*	UDP-glycosyltransferase	Catalyzes developmentally regulated glycosylation of a bitter cucurbitacin intermediate	Validated	Enzyme assay, heterologous expression, metabolite analysis	[5]
*C. sativus*	*CsMATE1*	MATE/DTX transporter	Mediates vacuolar sequestration of cucurbitacin C in leaf mesophyll cells	Validated	Transport assay, subcellular localization, genetic and metabolite analysis	[6]
*Cucumis melo*	*CmBi*	Oxidosqualene cyclase	Catalyzes cucurbitadienol formation in cucurbitacin B biosynthesis	Validated	Heterologous expression and pathway reconstruction	[20]
*C. melo*	*CmBt*	bHLH transcription factor	Predominantly regulates cucurbitacin B biosynthesis in leaves and cooperates with CmBr	Validated	CRISPR/Cas9, protein interaction, promoter activation	[20,54]
*C. melo*	*CmBr*	bHLH transcription factor	Regulates cucurbitacin B accumulation in fruits and roots and contributes to CPPU-induced bitterness	Validated	CRISPR/Cas9, expression analysis, protein interaction, metabolite quantification	[21,54]
*C. melo*	*CmRSM1*	MYB-related transcription factor	Activates CmBr expression and promotes CPPU-induced fruit bitterness	Validated	Promoter binding, dual-luciferase assay, genetic analysis	[21]
*C. melo*	*CmWRKY13*	WRKY transcription factor	Activates cucurbitacin B biosynthetic genes and interacts with CmBt	Supported	Promoter binding, transcriptional activation, protein interaction	[22]
*Luffa acutangula*	*LaBi*	Oxidosqualene cyclase	Candidate scaffold-forming enzyme associated with cucurbitacin biosynthesis	Candidate	Transcript–metabolite correlation and promoter regulation; catalytic product not directly confirmed	[23]
*L. acutangula*	*LaAREB1*	AREB/ABF transcription factor	Binds and activates the *LaBi* promoter in ABA-related signaling	Supported	Yeast one-hybrid and dual-luciferase assays	[23]
*Hemsleya macrosperma*	*HmSE1*	Squalene epoxidase	Produces 2,3-oxidosqualene and contributes to precursor supply for cucurbitacin IIa biosynthesis	Validated	Biochemical characterization, heterologous expression, expression–metabolite association	[24]
*Hemsleya chinensis*	*HcSE1*	Squalene epoxidase	Enhances 2,3-oxidosqualene supply for cucurbitacin IIa pathway reconstruction	Validated	Heterologous expression and product-based pathway analysis	[25]
*H. chinensis*	*HcSE2*	Squalene epoxidase	Contributes to precursor supply in reconstructed cucurbitacin IIa biosynthesis	Validated	Heterologous expression and product-based pathway analysis	[25]
*H. chinensis*	*HcOSC6*	Oxidosqualene cyclase	Catalyzes cucurbitadienol formation	Validated	Heterologous expression, site-directed mutagenesis, overexpression, CRISPR/Cas9 editing	[58,62]
*H. chinensis*	*HcCYP81Q59*	Cytochrome P450 monooxygenase	Catalyzes an additional oxidation step in cucurbitacin IIa biosynthesis	Validated	Yeast and *N. benthamiana* pathway reconstruction with product identification	[25]
*H. chinensis*	*HcAT1*	Acyltransferase	Catalyzes an acetylation step associated with cucurbitacin IIa formation	Validated	Heterologous pathway reconstruction and product analysis	[25]
*Aquilaria sinensis*	*As03G2784*	Bi-like oxidosqualene cyclase	Supported scaffold-forming enzyme in a woody plant	Supported	Transient expression in *N. benthamiana* and metabolite detection	[13]
Cucurbit crops	*Brp*	bHLH transcription factor	Candidate regulator potentially associated with cucurbitacin-related traits	Candidate	Comparative genomics, phylogenetic clustering, expression association	[26]

Evidence levels: Validated—direct genetic, biochemical, transport, or product-based functional evidence; Supported—promoter binding/transactivation, transient expression, or pathway reconstruction without native genetic confirmation; Candidate—genomic, phylogenetic, co-expression, or correlation-based assignment. CPPU, N-(2-chloro-4-pyridyl)-N′-phenylurea (forchlorfenuron).

## 4. Molecular Regulation of Cucurbitacin Biosynthesis

Cucurbitacin accumulation is controlled by regulatory networks that determine the timing, location, and intensity of biosynthetic-gene expression [19,20,21,22,23,26,54]. Current evidence supports an emerging combinatorial model in which tissue-preferential basic helix–loop–helix (bHLH) transcription factors form the principal regulatory layer, while developmental programs, hormonal signals, environmental conditions, and accessory regulators modify pathway activity [19,20,21,22,23,26,54]. Transport and metabolite turnover further influence the final cucurbitacin profile [5,6,55]. The evidence categories used here follow those defined in Section 3.5 and Table 1. This distinction is important because transcriptomic correlation, promoter-motif prediction, or genomic proximity can identify regulatory candidates but does not establish causal regulation.

### 4.1. The Central Regulatory Role of bHLH Transcription Factors

Comparative genomics supports the importance of bHLH transcription factors in the spatial regulation of cucurbitacin biosynthesis. Analysis of 1160 bHLH genes from seven cucurbit crops revealed conserved bitterness-associated transcription-factor clusters together with lineage-specific duplication and divergence. A paralogous cluster containing the candidate root-associated regulator *Brp* was also identified, suggesting that duplication of ancestral *bHLH* genes contributed to the development of partially independent regulatory modules in fruits, leaves, and roots [26].

This organization provides a molecular explanation for why cucurbitacin accumulation can differ among organs within the same plant. Closely related bHLH proteins may recognize similar E-box elements but differ in expression pattern, DNA-binding efficiency, interaction partners, and target-gene specificity. Tissue-preferential regulation should therefore be interpreted as a quantitative and partially overlapping system rather than as a set of completely independent switches.

The evolutionary expansion of bitterness-associated *bHLH* genes helps explain organ-specific cucurbitacin accumulation and the retention of vegetative defense during fruit domestication. However, organ-preferential regulation does not by itself establish a suitable target for crop improvement. Reducing the activity of a fruit-preferential regulator may improve edible quality, but effects in roots and leaves, as well as consequences for both generalist and specialist herbivores, must be evaluated before an agronomic benefit can be inferred.

### 4.2. Cooperative and Combinatorial Regulation

Recent genetic evidence demonstrates that cucurbitacin regulation can involve cooperation between related bHLH proteins. In melon, CRISPR/Cas9 analysis showed that *CmBt* predominantly regulates cucurbitacin B accumulation in leaves, whereas its paralog *CmBr* has stronger effects in fruits and roots. The CmBt and CmBr proteins physically interact and synergistically activate the scaffold-forming gene *CmBi*. Double mutants display a greater reduction in cucurbitacin B than either single mutant and completely prevent forchlorfenuron (CPPU)-induced fruit bitterness [54].

These findings revise the simple model in which each regulator controls only one organ. Instead, cucurbitacin biosynthetic genes appear to integrate overlapping inputs from several transcription factors. Final pathway activity may depend on their relative abundance, dimerization, promoter affinity, and interactions with additional proteins.

Accessory regulators provide further evidence for combinatorial control. In melon, CmRSM1 binds to and activates the *CmBr* promoter and contributes to CPPU-induced bitterness [21]. CmWRKY13 activates several cucurbitacin B biosynthetic genes and interacts with the CmBt protein [22]. In *Luffa acutangula*, LaAREB1 binds to and activates the promoter of the candidate scaffold-forming gene *LaBi*, linking abscisic acid-related signaling with pathway regulation [23]. However, the evidence levels differ among these factors. The role of CmRSM1 is supported by promoter-binding, transactivation, and genetic analyses, whereas the assignments of CmWRKY13 and LaAREB1 rely mainly on promoter assays and protein-interaction evidence without comprehensive native loss-of-function validation. Moreover, because the catalytic function of LaBi remains incompletely established, activation of its promoter cannot alone demonstrate regulation of cucurbitacin biosynthesis.

Evidence for direct regulation should therefore include more than co-expression or motif prediction. Strong regulatory assignments require genetic phenotypes together with promoter binding, transcriptional activation, chromatin occupancy, or protein-interaction evidence. Factors supported only by transcriptome networks or promoter predictions should be described as candidates rather than confirmed regulators.

### 4.3. Developmental, Hormonal, Environmental, and Herbivore-Induced Regulation

Cucurbitacin abundance changes substantially during fruit development. In melon, bitter genotypes showed high cucurbitacin B concentrations and elevated expression of biosynthetic genes during early fruit development, followed by a progressive decline during maturation. Non-bitter genotypes maintained low metabolite and transcript levels throughout development [51].

More recent transcriptomic and metabolomic analyses of melon germplasm with contrasting cucurbitacin B decline patterns showed that developmental changes involve more than transcriptional control of biosynthesis. Differences in hormone signaling, senescence, terpenoid metabolism, growth allocation, and ATP-binding cassette (ABC) transporter expression were associated with cucurbitacin B accumulation and clearance. Eleven ABC transporter genes were strongly correlated with cucurbitacin B dynamics [55]. These correlations identify candidates for cucurbitacin transport or turnover but do not demonstrate transporter function; direct transport assays, subcellular localization, and genetic validation are still required.

Plant-growth regulators can disturb this developmental balance and induce undesirable fruit bitterness. Current genetic evidence indicates that the response to CPPU depends on the combined activity of the CmBt–CmBr module [54]. This provides a direct connection between an external growth-regulator treatment, tissue-specific transcriptional regulation, and cucurbitacin accumulation.

Direct metabolite measurements demonstrate that cucurbitacin accumulation can be induced in particular experimental systems but that this response is not universal. Feeding by *Tetranychus urticae* increased cucurbitacin C concentrations in bitter cucumber [43], whereas leaf damage increased cucurbitacin B and D concentrations in zucchini [52]. In contrast, McCloud et al. detected no cucurbitacin accumulation inside or outside the feeding trenches produced by *Epilachna borealis* [53]. These contrasting findings distinguish evidence for the occurrence of induction from evidence for its underlying mechanism. The causal sequence linking herbivore perception, hormonal signaling, transcriptional activation, metabolite accumulation, and subsequent effects on herbivore performance remains incompletely resolved.

### 4.4. Natural Variation, Domestication, and Comparative Gaps

Natural variation in transcription-factor genes and their regulatory regions has contributed substantially to differences in cucurbitacin accumulation among genotypes. Gene duplication and divergence generated organ-preferential regulatory modules, while crop selection favored alleles that reduced bitterness in edible fruits. This regulatory modification was more advantageous than eliminating the entire pathway because defensive capacity could be retained in vegetative or underground organs.

Recent melon research illustrates both the potential value and the limitations of regulatory variation. Disruption of both *CmBt* and *CmBr* eliminates CPPU-induced fruit bitterness, identifying these loci as potential targets for stabilizing fruit quality [54]. However, simultaneous suppression across tissues could reduce deterrence against generalist herbivores while also decreasing attraction or feeding stimulation in cucurbitacin-adapted specialists. Consequently, the agronomic outcome cannot be predicted from fruit bitterness alone. Evaluation should include cucurbitacin profiles in fruits, leaves, and roots together with responses of representative generalist and specialist herbivores under field-relevant conditions.

Regulatory evidence outside Cucurbitaceae remains limited. In agarwood-forming *Aquilaria* tissues, red- and far-red-light treatments were associated with changes in cucurbitacin yield, transcript abundance, small-RNA profiles, and DNA methylation [56]. These results support possible light-responsive and epigenetic regulation of cucurbitane-type metabolism, but the relevant transcription factors, direct target genes, and causal relationships have not been established. Homologs of the tissue-preferential bHLH regulators characterized in cucumber and melon have not been functionally validated in woody plants, and it remains unknown whether wounding, microbial infection, wood formation, or long-term development directly activates cucurbitacin biosynthesis. Woody plants should therefore be treated as emerging comparative systems rather than as evidence for a conserved regulatory network across plant lineages.

### 4.5. Current Regulatory Model and Evidence Limits

Available evidence supports a combinatorial model of cucurbitacin regulation [19,20,21,22,23,26,54]. Tissue-preferential bHLH factors provide the principal characterized transcriptional input, while related bHLH proteins may cooperate through physical interaction and shared target promoters [19,20,26,54]. Developmental, hormonal, environmental, and herbivore-associated signals can further modify pathway activity [21,22,23,43,51,52,53,55,56]. Transport, sequestration, metabolic turnover, and resource allocation subsequently shape the final cucurbitacin profile [5,6,55].

However, evidence beyond the core bHLH regulators remains uneven. Several accessory regulators, environmental inputs, and transport-associated genes are supported mainly by promoter assays, expression correlations, or metabolite associations, and their positions in causal regulatory networks therefore remain provisional.

## 5. Contrasting Ecological Consequences of Cucurbitacins in Plant–Herbivore Interactions

**Figure 4:**
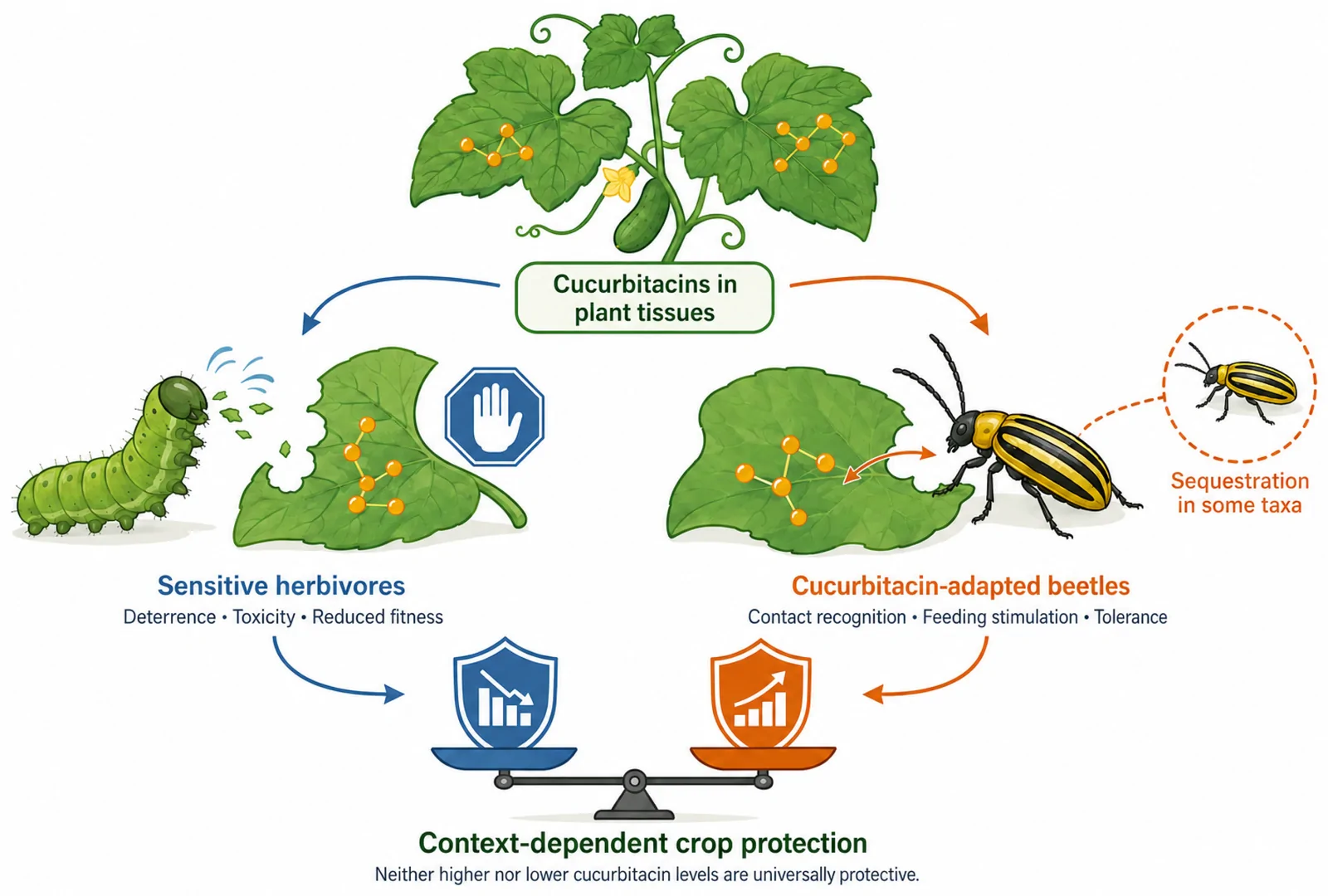
Herbivore-dependent ecological consequences of cucurbitacins. Cucurbitacins can deter or reduce fitness in sensitive herbivores, whereas adapted beetles can recognize them as contact feeding cues, tolerate them, and sometimes sequester them. Their crop-protection effects are therefore context-dependent. Orange symbols denote cucurbitacins, and the dashed inset indicates taxon-dependent sequestration.

Cucurbitacins are widely regarded as defensive specialized metabolites because their bitterness and toxicity can reduce host acceptance and impair the performance of many cucurbitacin-sensitive herbivores [7,27,28,29,30,64]. From the plant perspective, these deterrent and toxic effects constitute a defensive function. By contrast, contact recognition, arrestment, and feeding stimulation in cucurbitacin-adapted diabroticite beetles reflect herbivore adaptations that exploit plant chemical cues, rather than a second function of the plant metabolite. Some adapted beetles can also metabolize or sequester cucurbitacins after ingestion. The ecological outcome therefore depends on compound identity and concentration, tissue distribution, plant genotype, herbivore identity, sensory perception, physiological tolerance, and post-ingestive metabolism. These contrasting consequences and their implications for crop protection are summarized conceptually in Figure 4, whereas the representative experimental systems, reported responses, and evidence levels are compiled in Table 2.

### 5.1. Constitutive Defense and Domestication

Constitutive cucurbitacins are present before herbivore attack and can immediately influence insect host selection, feeding, survival, growth, and reproduction [27,32,33,64,65,66]. Their accumulation in roots, cotyledons, young leaves, and other vulnerable tissues is consistent with a defensive role during early plant development [6,32,33,40].

Comparisons between wild and domesticated squash provide direct evidence that crop selection has altered constitutive cucurbitacin-based defense. Wild *Cucurbita argyrosperma* populations contained substantially higher cucurbitacin concentrations in roots and cotyledons than domesticated varieties and also showed higher expression of several pathway-associated genes [32]. Larvae of *Diabrotica balteata* and *Acalymma* spp. preferred wild roots, but the high-cucurbitacin tissues reduced the performance of *D. balteata* while having little negative effect on the more cucurbitacin-tolerant *Acalymma* spp. [32].

Independent domestication events in *C. pepo* also generated marked differences in cotyledon cucurbitacin accumulation and preference of cucurbitacin-adapted beetles. Genetic mapping identified a major locus containing candidate regulatory and transport genes rather than only core biosynthetic enzymes, indicating that domestication can reshape chemical defense through changes in regulation and metabolite distribution [33].

These findings demonstrate that domestication-related reductions in cucurbitacin accumulation do not have a uniform ecological consequence. Reduced accumulation may weaken resistance to cucurbitacin-sensitive herbivores while diminishing contact-recognition or feeding cues used by adapted beetles. Domestication-related loss of bitterness should therefore not be interpreted simply as a general decline in plant defense.

### 5.2. Herbivore-Induced and Systemic Responses

Evidence for herbivore- or damage-induced cucurbitacin accumulation is system-dependent. Feeding by *Tetranychus urticae* increased cucurbitacin C concentrations in bitter cucumber [43], whereas leaf damage increased cucurbitacins B and D in zucchini [52]. In contrast, no accumulation was detected inside or outside feeding trenches produced by *Epilachna borealis* [53]. Feeding by *D. balteata* produced cultivar- and organ-specific changes in defense-associated gene expression and trichome production in *C. argyrosperma* [69], while another cucumber study detected only minor or variety-dependent changes in cucurbitacin accumulation [31]. Similarly, aboveground herbivory reduced subsequent root preference by *Acalymma vittatum* larvae without increasing cucurbitacin C concentrations in cucumber leaves or roots [70]. Thus, changes in gene expression, physical defenses, biomass, or herbivore preference should not be interpreted as cucurbitacin induction without a quantitative increase in the metabolites themselves.

For the narrow claim of cucurbitacin induction, time-resolved quantitative evidence of an increase following herbivory is sufficient. A stronger claim that induction enhances plant resistance additionally requires evidence of altered herbivore behavior or performance, whereas a mechanistic claim requires links among perception, signaling, pathway regulation, and metabolite accumulation. These three levels of inference should not be conflated.

### 5.3. Deterrent and Toxic Effects on Cucurbitacin-Sensitive Herbivores

For many cucurbitacin-sensitive herbivores, cucurbitacins act as feeding deterrents, oviposition deterrents, toxins, or developmental inhibitors. Choice assays using cucurbitacin B demonstrated reduced feeding by several beetles and lepidopteran larvae and reduced oviposition by *Ostrinia nubilalis* and *Spodoptera exigua* [64]. However, responses were not uniform among feeding guilds, because some sap-feeding insects showed neutral or positive responses to cucurbitacin-treated substrates.

Chemical structure strongly influences biological activity. Comparative assays using modified cucurbitacin derivatives showed that relatively small structural changes produced species-specific shifts in antifeedant effects. Compounds that deterred a polyphagous noctuid did not necessarily deter a cucurbit-associated specialist, and one modified derivative stimulated specialist feeding [7].

Therefore, results obtained with one purified cucurbitacin cannot be generalized to the entire compound class. Hydroxylation, acetylation, hydrogenation, oxidation, and glycosylation may alter receptor activation, membrane transport, metabolic stability, tissue distribution, and toxicity. Studies should report the exact compound, purity, concentration, application method, and conjugation state and should distinguish behavioral deterrence from post-ingestive toxicity.

### 5.4. Contact Recognition and Feeding Stimulation in Cucurbitacin-Adapted Beetles

In diabroticite beetles, cucurbitacins act as active feeding stimulants rather than merely compounds that fail to deter feeding. Comparative analyses of *Cucurbita* species revealed a positive relationship between tissue cucurbitacin content and the arrestment and feeding intensity of southern and western corn rootworms [65]. Analysis of 19 species and 46 cultivars from *Cucurbita*, *Cucumis*, and *Citrullus* further demonstrated that cotyledon cucurbitacin composition and concentration strongly influenced beetle feeding behavior [66].

The sensory basis of this response has been investigated in adult western corn rootworm. Chemosensory hairs on the maxillary galea responded electrophysiologically to cucurbitacin B at concentrations as low as 0.1 μM. Bilateral removal of these sensilla substantially reduced cucurbitacin-stimulated feeding, providing direct evidence that peripheral gustatory structures mediate recognition [67].

Cucurbitacins are nevertheless only one component of host selection. Volatile and non-volatile host cues, tissue characteristics, and plant developmental context can also influence orientation and feeding before or after contact [34,35,65,66,67]. Cucurbitacins are therefore best interpreted as potent contact-based arrestants and phagostimulants within a broader host-recognition system.

This sensory adaptation explains why high constitutive cucurbitacin concentrations may suppress sensitive herbivores while increasing feeding by adapted beetles. Domestication-associated reductions in cucurbitacin levels decreased root preference by *D. balteata* and *Acalymma* spp. in *C. argyrosperma* [32], whereas variation among independently domesticated *C. pepo* lineages altered *A. vittatum* preference [33].

### 5.5. Metabolic Transformation and Sequestration

Adapted beetles can absorb, transform, and retain cucurbitacins after ingestion. Feeding experiments with purified cucurbitacin D showed that *Diabrotica virgifera virgifera* and *D. undecimpunctata howardi* produced acetylated and non-acetylated glucosides through glucosylation, hydrogenation, desaturation, and acetylation reactions [68]. Several metabolites persisted after the insects were transferred to a cucurbitacin-free diet, supporting true sequestration rather than temporary retention in the digestive tract.

These transformations may reduce chemical reactivity, facilitate transport, alter toxicity, or enable storage in less sensitive tissues. However, the specific insect enzymes, transporters, storage proteins, and cellular compartments responsible remain largely unknown.

Metabolic transformation also complicates ecological interpretation because the compound detected in an insect may differ from the compound originally consumed. The stored derivative may also have biological properties different from those of the plant precursor. Future studies should therefore distinguish compounds in plant tissues, gut contents, haemolymph, excreta, and storage tissues.

Sequestration does not necessarily provide protection against natural enemies. In a belowground tritrophic experiment, *D. balteata* larvae feeding on high-cucurbitacin wild squash were not better protected against a predatory rove beetle. Larvae feeding on cucurbitacin-free domesticated roots were attacked less frequently, suggesting that the physiological cost of processing cucurbitacins may offset potential defensive benefits [71]. Similarly, cucurbitacins acquired by *D. balteata* larvae provided little consistent protection against predators, entomopathogenic nematodes, fungi, or bacteria [31].

The ecological value of sequestration therefore depends on compound identity, concentration, insect life stage, storage tissue, and natural-enemy species. Detection of cucurbitacins in an insect should not automatically be interpreted as evidence of effective acquired defense.

### 5.6. Ecological Trade-Offs and Crop-Protection Implications

Exploitation of cucurbitacins by adapted beetles does not represent an evolutionary reversal of the plant chemical-defense trait. Instead, plant defense and herbivore adaptation coexist. Spatial and temporal variation in the relative abundance of cucurbitacin-sensitive and cucurbitacin-adapted herbivores may impose opposing selection on cucurbitacin production and could contribute to balancing selection. Accordingly, increasing cucurbitacin levels may suppress sensitive herbivores while enhancing contact recognition and feeding by adapted beetles (Figure 4).

These contrasting ecological consequences also influence pest management applications. Cucurbitacins can be used as feeding stimulants in attract-and-kill systems targeting diabroticite beetles. A multicomponent system combining the aggregation pheromone vittatalactone, watermelon-derived host cues, and cucurbitacin E glycoside increased attraction and feeding by striped and spotted cucumber beetles, although trap design and placement strongly affected field performance [72].

However, cucurbitacin-based formulations are not consistently effective. In field trials, adding a cucurbitacin-containing gustatory stimulant to spinosad or carbaryl did not reliably improve cucumber beetle control in melon [73]. Formulation stability, compound identity, toxin compatibility, release rate, beetle physiological state, pest density, bait placement, and competing plant cues may all influence efficacy.

Resistance and tolerance are complementary but distinct strategies. In wild *Cucurbita pepo* subsp. *texana*, the effects of foliar damage on growth and reproduction depended on damage intensity and spatial pattern [74]. Across six cucurbit taxa, resistance and tolerance also differed between foliar and root herbivory and did not vary consistently with domestication [75]. Because tolerance is a whole-plant response, it is considered here as agronomic context rather than as a cucurbitacin-specific mechanism.

Uniform selection for higher or lower total cucurbitacin content is therefore unlikely to provide robust resistance. Tissue-specific manipulation should be considered only for defined pest communities, while resistance to adapted beetles may depend more strongly on additional traits, including mucilaginous sap and trenching responses [53], trichomes and other physical defenses [69], volatile cues, and whole-plant tolerance. Candidate genotypes should be evaluated against both cucurbitacin-sensitive and cucurbitacin-adapted herbivores under field-relevant conditions within an integrated pest management framework. Thus, cucurbitacin concentration alone is not a reliable target for insect-resistance breeding.

## 6. Applications in Crop Improvement and Sustainable Pest Management

The contrasting effects of cucurbitacins create both opportunities and constraints for crop improvement. From an agroecological perspective, neither increasing nor decreasing cucurbitacin concentration is expected to provide universally effective resistance: higher concentrations may deter generalist or non-adapted herbivores while enhancing host recognition and feeding by cucurbitacin-adapted specialists, whereas lower concentrations may produce the opposite outcome. Cucurbitacin-based improvement must therefore begin with a clearly defined agronomic objective, target-pest community, plant tissue, developmental stage, and production environment. Application-oriented research should focus on the identity, location, and timing of cucurbitacin accumulation rather than simply increasing or decreasing total content.

### 6.1. Breeding Objectives and Natural Variation

A context-dependent breeding objective may combine stable suppression of bitterness in edible fruits with retention of selected cucurbitacin profiles in non-edible tissues. However, this phenotype should not be assumed to confer overall insect resistance. Candidate germplasm must be tested against both generalist herbivores and specialist diabroticite beetles because elevated vegetative cucurbitacin concentrations may reduce feeding by the former while increasing host recognition or feeding stimulation in the latter. Breeding objectives should therefore be defined for the locally important pest complex rather than for cucurbitacin concentration alone.

Wild relatives and traditional landraces provide important sources of variation in cucurbitacin composition, tissue specificity, and developmental regulation. Modern pangenomic resources can reveal structural variants, gene presence–absence variation, copy-number changes, and regulatory polymorphisms that are absent from a single reference genome. A pangenome represents the combined core and accessory genomic variation identified across multiple accessions of a species, whereas a super-pangenome extends this framework across multiple related species or an entire genus. A graph-based melon pangenome demonstrated that structural variation can substantially improve the resolution of resistance loci and trait-associated regions [76]. A telomere-to-telomere super-pangenome encompassing all seven *Citrullus* species further identified extensive genomic variation associated with watermelon domestication, fruit bitterness, quality, and disease resistance [77].

These resources provide a foundation for marker-assisted and genomic selection. Nevertheless, markers should be linked to targeted metabolite profiles and insect phenotypes rather than sensory bitterness alone. Different cucurbitacin derivatives may have distinct ecological effects even when overall fruit bitterness is similar. Validation should include replicated measurements of individual cucurbitacins in edible and vegetative tissues, plant performance, and responses of representative generalist and specialist herbivores under field-relevant conditions. Natural variation in other defensive traits—including trichomes, mucilaginous sap, volatile terpenes, phenology, and damage tolerance—should also be considered rather than treating cucurbitacins as the sole resistance determinant.

### 6.2. Genome Editing and Precision Regulation

On the basis of current evidence, genome editing appears more readily justified for stabilizing fruit quality or testing regulatory mechanisms than for producing generalized insect resistance through uniform changes in cucurbitacin concentration [21,54,78,79]. Complete disruption of a core biosynthetic enzyme could eliminate cucurbitacins from multiple tissues, improving palatability but potentially increasing susceptibility to generalist herbivores. Conversely, increasing pathway activity could enhance deterrence against some pests while increasing feeding by adapted specialists. More restricted targets, such as tissue-preferential transcription factors, experimentally validated promoter elements, transporters, or branch-specific modifying enzymes, may provide greater spatial or chemical precision, but they do not remove this ecological trade-off.

Recent cucumber research illustrates the complexity of such regulatory targets. The AP2-like regulator *NPF1* affects both parthenocarpy and fruit bitterness by suppressing *Bt* expression and cucurbitacin C biosynthesis [78]. This pleiotropic relationship shows that modifying a bitterness regulator may also alter fruit development or other agronomic traits. Consequently, the value of an editing target cannot be inferred solely from reduced bitterness or altered cucurbitacin content.

Promoter editing may offer greater precision than complete gene knockout. Modification of tissue-preferential enhancers, bHLH-binding motifs, or hormone-responsive elements could adjust the timing and intensity of pathway activation. Multiplex editing may also be required where paralogous regulators have overlapping functions. Emerging CRISPR systems, base editing, prime editing, and DNA-free delivery methods broaden the available strategies, although regeneration efficiency, genotype dependence, chimerism, pleiotropy, and off-target effects remain important limitations [79]. Until the relevant regulatory elements and their tissue specificity have been causally validated, promoter editing should be regarded primarily as an experimental tool rather than an immediately deployable breeding solution.

Edited lines should be evaluated across roots, leaves, flowers, and fruits and under contrasting environmental conditions. Measurements should include individual cucurbitacins, crop growth, yield, fruit quality, and responses to both generalist and specialist herbivores. Multi-season field trials should additionally assess genotype-by-environment interactions, the stability of fruit bitterness, natural-enemy and pollinator activity, pathogen incidence, and possible compensatory changes in other defense metabolites.

### 6.3. Metabolic Engineering, Behavioral Control, and Pest-Targeted Biotechnology

The immediate value of metabolic engineering lies mainly in pathway reconstruction, enzyme validation, production of analytical standards, and generation of defined compounds for ecological assays. Translation to intact crops is more difficult because precursor supply, scaffold formation, oxidative tailoring, terminal modification, transport, sequestration, and metabolic turnover must be coordinated. Increasing pathway flux without appropriate compartmentation may cause intermediate toxicity, metabolic imbalance, or unintended changes in plant development and defense.

Heterologous systems remain valuable for identifying enzyme function and clarifying whether particular structural modifications alter deterrence, toxicity, or feeding stimulation. However, engineered yeast, tobacco, or hairy-root cultures—transformed root cultures commonly induced by *Rhizobium rhizogenes*—cannot fully reproduce the tissue distribution, developmental regulation, or multi-trophic interactions occurring in intact crop plants [25,59,63]. Their results should therefore support biochemical interpretation rather than be treated as direct evidence of agronomic performance [25,59,63].

The economic importance of diabroticite beetles provides a practical rationale for developing ecologically informed management methods. The western corn rootworm, *Diabrotica virgifera virgifera*, alone has been estimated to cause more than USD 1 billion annually in yield losses and management costs in the United States [37,80]. This estimate primarily concerns maize rather than cucurbit production and should not be interpreted as a direct estimate of cucurbit-crop losses. Nevertheless, it demonstrates the economic importance and management adaptability of cucurbitacin-responsive diabroticites and supports the evaluation of behavioral control methods within integrated programs.

The attraction of diabroticite beetles provides a separate application in behavioral pest management. Cucurbitacins can act as short-range feeding stimulants when combined with pheromones, host volatiles, or insecticides. This strategy exploits a sensory and behavioral adaptation of specialist beetles rather than altering cucurbitacin concentrations within the crop itself. Its effectiveness depends on formulation stability, toxicant compatibility, release rate, bait placement, pest density, competing host cues, and the immigration of beetles from surrounding habitats. Cucurbitacin-based attract-and-kill systems should therefore complement crop rotation, resistant cultivars, biological control, and pest monitoring rather than function as stand-alone interventions.

RNA interference (RNAi) provides another pest-targeted strategy that does not require manipulation of plant cucurbitacin content. Crops expressing double-stranded RNAs (dsRNAs), or externally applied dsRNA formulations, may suppress essential genes in target insects and could potentially be combined with behavioral baits or other integrated pest management tactics [81]. However, variation in insect RNAi sensitivity, dsRNA delivery and persistence, production cost, resistance evolution, and field stability currently limits broad application. RNAi should therefore be considered a complementary technology whose performance must be compared with existing cultural, biological, and chemical control methods.

### 6.4. Agroecological, Agronomic, and Risk Evaluation

Manipulating cucurbitacins may produce unintended trade-offs. Increased accumulation could suppress aphids, mites, or non-adapted chewing insects while increasing attraction to specialist beetles. Reducing cucurbitacins may improve fruit quality but weaken resistance in vegetative tissues. Changes in plant chemistry may also affect natural enemies, pollinators, pathogens, and soil organisms. These indirect effects cannot be predicted reliably from purified-compound toxicity assays and require evaluation in intact plants and multi-trophic field systems.

Food quality and safety are central constraints because cucurbitacins are intensely bitter and biologically active. Breeding or editing programs must ensure stable suppression in edible organs under drought, temperature variation, nutrient stress, and plant-growth-regulator treatments. Potential reversion to fruit bitterness, variation among genetic backgrounds, effects on yield and reproductive development, and compliance with jurisdiction-specific food-safety and biotechnology regulations must also be considered.

Environmental risk assessment is equally important for pest-targeted biotechnologies. For RNAi-based crops or biopesticides, evaluation should include dsRNA degradation and environmental persistence, exposure of non-target organisms, sequence-dependent off-target effects, resistance development, human and animal safety, and post-release monitoring [82]. Comparable assessment is required for cucurbitacin-containing baits, particularly with respect to toxicant exposure, attraction of non-target beetles, environmental persistence, and interactions with pollinators and natural enemies.

Applications should therefore be evaluated within an integrated pest management framework. Field trials should simultaneously assess metabolite profiles, crop quality, yield, target-pest resistance, specialist attraction, natural-enemy activity, and environmental stability. Two contrasting but potentially complementary improvement approaches should be compared. A molecular-breeding approach may prioritize stable fruit quality and carefully restricted tissue-specific regulation, whereas an agroecological approach may combine pest monitoring, economic thresholds, crop rotation, biological control, behavioral baits, damage tolerance, and resistance traits that do not depend on cucurbitacins.

The most useful phenotype will not necessarily contain the highest or lowest cucurbitacin concentration. Indeed, manipulating cucurbitacin concentration alone is unlikely to provide a broadly effective insect-resistance strategy because either direction may favor a different herbivore group. The most defensible near-term applications are therefore likely to involve preventing undesirable bitterness in edible tissues, using cucurbitacins as behaviorally active components of targeted baits, and integrating cucurbitacin-associated traits with additional plant defenses and agroecological management practices.

## 7. Current Challenges and Future Perspectives

Despite major advances in cucurbitacin biology, current knowledge remains concentrated in a small number of cucurbit crops and individual compounds. Complete biosynthetic routes, transport processes, regulatory networks, insect-adaptation mechanisms, and field-level consequences remain incompletely resolved. Rather than reiterating the candidate genes and biotechnological approaches discussed in previous sections, four priorities emerge from the current evidence: (1) converting pathway and transporter candidates into experimentally validated components; (2) establishing causal links among herbivory, cucurbitacin responses, and herbivore performance; (3) improving analytical standardization and spatial resolution; and (4) testing molecular predictions in comparative and field-based agroecological contexts. Identification of additional candidates without functional or ecological validation should be considered a lower priority.

### 7.1. Priority 1: Completing and Validating Biosynthetic and Transport Networks

As detailed in Section 3.5 and Table 1, the primary biosynthetic priority is to convert unvalidated terminal-tailoring candidates into product-defined pathway components.

Transport represents a related but distinct knowledge gap. Cucurbitacins may be synthesized and stored in different cell types, transported between organs, or converted between aglycone and conjugated forms. Although ATP-binding cassette (ABC) and multidrug and toxic compound extrusion (MATE) transporters have been proposed from expression correlations, the substrate specificity and physiological function of most candidates have not been demonstrated. Similar challenges occur in other plant terpenoid pathways, in which intracellular trafficking, vacuolar sequestration, and long-distance transport remain incompletely resolved [83].

Direct transport assays, subcellular localization, tissue-specific genetic manipulation, and simultaneous measurement of intracellular and extracellular metabolites are therefore required. Resolving these processes is necessary not only for reconstructing complete pathways but also for determining whether bitterness in edible organs can be altered independently of cucurbitacin accumulation in vegetative tissues.

### 7.2. Priority 2: Establishing Causal Regulatory and Ecological Mechanisms

Omics analyses have generated numerous candidate regulatory genes, but few have been incorporated into experimentally supported causal networks. Future studies should connect genetic manipulation with direct promoter occupancy, transcriptional activation or repression, protein interactions, and quantitative changes in individual cucurbitacins. Chromatin immunoprecipitation sequencing (ChIP-seq), cleavage under targets and tagmentation (CUT&Tag), promoter-reporter assays, and native genetic analyses should be used selectively to test defined regulatory hypotheses rather than to generate additional unvalidated networks.

As summarized in Section 2.4 and Section 5.2, herbivore-induced cucurbitacin accumulation is context-dependent rather than universal. Future studies should identify the genetic, biotic, tissue-specific, and environmental determinants of this response and separately test metabolite induction, its signaling basis, and its consequences for herbivore preference and performance.

Such experiments should distinguish insect feeding from mechanical damage and should measure early signaling events, biosynthetic-gene expression, individual metabolites, and herbivore performance within the same time series. The phrase “plant regulation” should be avoided unless the specific process is identified; relevant processes may include herbivore perception, hormonal signaling, transcriptional activation, biosynthesis, transport, sequestration, and metabolic turnover. Plant specialized metabolites should therefore be examined as mediators of multi-trophic interactions rather than as isolated toxins [84].

Mechanisms of insect adaptation require similar attention. Attraction and feeding stimulation in specialist beetles should be interpreted as herbivore adaptations to a plant defensive metabolite, rather than as a second defensive function of cucurbitacins for the plant. Direct evidence remains limited for the gustatory receptors, neural pathways, detoxification enzymes, transporters, and storage tissues that enable specialist beetles to exploit cucurbitacins. Comparative genomics, electrophysiology, isotope tracing, proteomics, and tissue-resolved metabolomics could identify the molecular basis of this adaptation.

However, molecular characterization alone will not predict the agronomic outcome. Experiments must also determine how the relative abundance of generalist and specialist herbivores, natural enemies, alternative hosts, plant tolerance, and environmental conditions modifies selection on cucurbitacin production. Paired assays involving representative generalist and specialist herbivores should therefore precede claims that a particular cucurbitacin phenotype confers crop resistance.

### 7.3. Priority 3: Standardization and Spatial Resolution

Comparison among studies is hindered by variation in extraction procedures, plant tissues, developmental stages, analytical platforms, reference standards, and reporting units. Total bitterness, total cucurbitacins, individual aglycones, and glycosides are not equivalent measurements.

A minimum reporting framework should specify plant genotype, tissue, developmental stage, growth environment, herbivory treatment, extraction method, analytical platform, calibration procedure, reporting unit, and the identities and concentrations of individual cucurbitacins. Standardized liquid chromatography–tandem mass spectrometry (LC–MS/MS) protocols, authentic standards, isotope-labelled internal standards, pooled quality-control samples, and shared spectral libraries would improve reproducibility and cross-study comparison.

Insect assays should distinguish host selection from post-ingestive effects. Choice tests are required to evaluate attraction, arrestment, and feeding preference, whereas no-choice assays are needed to assess toxicity, growth, development, survival, and reproduction. Studies based on purified compounds or artificial diets should clearly distinguish these results from responses observed on intact plants.

Spatial metabolomics and mass-spectrometry imaging provide promising approaches for mapping cucurbitacins within tissues and cell types [85]. Combining these methods with spatial transcriptomics or single-cell sequencing could determine whether biosynthetic genes, modifying enzymes, transporters, and final metabolites are co-localized or distributed among specialized cell populations. Nevertheless, spatial co-localization remains correlative unless supported by genetic perturbation, transport assays, or biochemical validation. The near-term goal should be a spatially resolved and evidence-graded model of cucurbitacin biosynthesis, modification, transport, and storage.

### 7.4. Priority 4: Comparative Validation and Agroecological Translation

Comparative studies should be extended to medicinal plants, wild species, and woody taxa in which cucurbitacins or related cucurbitane-type metabolites have been chemically detected. Priority should be given to phylogenetically informative lineages that can test whether cucurbitane-type pathways represent shared ancestry, independent evolutionary recruitment, or convergent assembly. Chemical detection alone should not be interpreted as evidence for conserved biosynthetic regulation or ecological function.

In non-cucurbit and woody plants, research should initially establish scaffold-forming enzymes and major downstream reactions before proposing complete pathways or regulatory networks. Ecological experiments should then determine whether the detected metabolites affect herbivores, pathogens, or other interacting organisms in intact plants. These systems are most valuable as comparative tests of pathway evolution rather than as immediate targets for metabolic engineering.

As discussed in Section 6.1, general pangenome frameworks [86] provide useful context, but cucurbit-specific conclusions should be supported by cucurbit genomic resources. Recently developed cucumber and watermelon graph-based pangenomes and super-pangenomes illustrate how population- and species-level structural variation can improve candidate-gene discovery and trait mapping [77,87]. However, these resources generate hypotheses; they do not establish gene function, metabolite phenotype, or insect resistance without experimental validation.

Laboratory and genomic evidence must ultimately be tested in realistic crop environments. A staged validation framework is recommended. First, controlled experiments should compare representative generalist and specialist herbivores on plants with quantitatively characterized cucurbitacin profiles. Second, multi-location and multi-season field trials should measure crop yield, fruit quality, pest-community composition, natural enemies, pollinators, pathogen incidence, environmental stress, and non-target effects. Third, validated information should be incorporated into integrated pest management decisions involving monitoring, economic thresholds, crop rotation, biological control, behavioral baits, and non-cucurbitacin resistance or tolerance traits.

The central objective should not be pathway enhancement or predictive manipulation of cucurbitacin concentration alone. Instead, research should establish a decision framework that determines whether cucurbitacin manipulation is agronomically justified, which compound and tissue should be targeted, which herbivore community is present, and when modification should be avoided. A successful research program may therefore conclude that stabilizing fruit quality, exploiting specialist behavior in targeted baits, or improving other defensive traits is more appropriate than breeding uniformly high- or low-cucurbitacin crops.

## 8. Conclusions

Cucurbitacins are structurally diverse triterpenoids that contribute to plant-specialized metabolism, fruit bitterness, domestication, and chemical defense. Their biosynthesis involves conserved precursor formation followed by lineage-specific scaffold formation, oxidative modification, terminal tailoring, transport, and tissue-preferential regulation. However, experimentally validated knowledge remains concentrated mainly in cucumber, melon, and watermelon. In many non-cucurbit and woody plants, current interpretations are still based primarily on chemical detection, genomic prediction, or transcriptomic association rather than complete functional validation.

From the plant perspective, the principal defensive consequences of cucurbitacins are deterrence, toxicity, or reduced performance in generalist and non-adapted herbivores. Attraction, arrestment, and feeding stimulation in specialist diabroticite beetles should instead be interpreted as herbivore adaptations involving cucurbitacin perception, tolerance, metabolism, or sequestration—not as a second defensive function of the plant trait. Cucurbitacin accumulation can also be induced by herbivory in particular experimental systems, although the underlying signaling mechanisms, taxonomic generality, and consequences for plant fitness remain to be established. Ecological outcomes therefore depend on compound identity, concentration, tissue distribution, developmental stage, and the composition of the local herbivore community.

These contrasting responses mean that neither uniformly increasing nor eliminating cucurbitacins is likely to provide broadly effective crop resistance. Reducing cucurbitacins in edible fruits may be justified for stabilizing crop quality and food safety, but retaining or increasing them in vegetative tissues cannot be assumed to improve resistance because this may simultaneously enhance host recognition and feeding by adapted specialists. Two complementary approaches should therefore be considered: molecular breeding and regulatory analysis directed primarily toward fruit quality and mechanistic understanding, and agroecological management integrating pest monitoring, economic thresholds, crop rotation, biological control, behavioral baits, plant tolerance, and defensive traits other than cucurbitacins. The most defensible applications are likely to be context-specific—preventing undesirable fruit bitterness, exploiting specialist behavior in targeted attract-and-kill systems, and incorporating cucurbitacin-associated traits into integrated pest management—rather than manipulating cucurbitacin concentration as a stand-alone insect-resistance strategy.

## Figures and Tables

**Table 2:** Summary of cucurbitacin-mediated effects on representative herbivores, including the experimental context in which each response was evaluated.

Herbivore Species	Feeding Guild/Adaptation	Plant Material or System	Cucurbitacin	Reported Response	Test System	Evidence Level	Reference
*Tetranychus urticae* (two-spotted spider mite)	Generalist herbivore	Intact *Cucumis sativus* plants and contrasting genotypes	Cucurbitacin C	Reduced feeding preference and mite performance; association with enhanced plant resistance	Intact-plant infestation, choice and performance assays, and metabolite–phenotype association	Supported	[27]
*Aphis gossypii* (melon aphid)	Generalist sap-feeding insect	Purified cucurbitacin B exposure and artificial-diet systems	Cucurbitacin B	Reduced survival, longevity, fecundity, and population growth; altered detoxification responses	Controlled feeding, artificial-diet, and demographic assays	Validated	[28,29]
*Spodoptera litura* (tobacco cutworm)	Polyphagous chewing herbivore	Purified compound isolated from *Citrullus colocynthis*	Cucurbitacin E	Feeding deterrence, mortality, and impaired larval development and reproduction	Purified-compound dose–response bioassays	Validated	[30]
*Ostrinia nubilalis* (European corn borer)	Generalist chewing herbivore	Cucurbitacin-treated feeding and oviposition substrates	Cucurbitacins	Reduced feeding acceptance and oviposition preference	Behavioral choice assays with treated substrates	Validated	[64]
*Spodoptera exigua* (beet armyworm)	Generalist chewing herbivore	Cucurbitacin-treated feeding substrates	Cucurbitacins	Feeding deterrence and reduced host acceptance	Behavioral feeding-preference assays with treated substrates	Validated	[64]
*Diabrotica balteata* (banded cucumber beetle)	Cucurbitacin-adapted diabroticite beetle	Intact cucurbit plants and purified-compound assays	Cucurbitacins B and C and derivatives	Feeding stimulation, host recognition, metabolic transformation, and sequestration	Plant-choice assays, purified-compound feeding, and insect metabolite profiling	Validated	[31,32]
*Diabrotica virgifera virgifera* (western corn rootworm)	Cucurbitacin-adapted diabroticite beetle	Purified compounds and cucurbit tissues	Cucurbitacins B and D	Gustatory stimulation, enhanced feeding, metabolism, and sequestration	Electrophysiology, sensillum-ablation feeding assays, and metabolite profiling	Validated	[65,66,67,68]
*Diabrotica undecimpunctata howardi* (southern corn rootworm)	Cucurbitacin-adapted diabroticite beetle	Purified cucurbitacin feeding assays	Cucurbitacin D and derivatives	Uptake, metabolic transformation, and sequestration	Controlled feeding followed by insect metabolite profiling	Validated	[68]
*Acalymma vittatum* (striped cucumber beetle)	Cucurbitacin-adapted cucurbit specialist	Intact *Cucurbita* species, subspecies, and cultivars	Cucurbitacins	Host recognition and feeding preference	Intact-plant and tissue-choice assays across genotypes	Supported	[33]

Evidence levels for herbivore responses: Validated—a behavioral, physiological, or metabolic response directly demonstrated under defined cucurbitacin exposure or through causal sensory or metabolic manipulation; Supported—an intact-plant, genotype, or metabolite–phenotype association in which cucurbitacin was not isolated as the sole causal factor.

## Data Availability

No new data were created or analyzed in this study. Data sharing is not applicable to this article.

## Supplementary Materials

The following supporting information can be downloaded at: https://www.mdpi.com/article/10.3390/plants15172660/s1, Table S1: Study-level evidence catalogue.
