# Supplementary material for: Cucurbitacins in Plant–Insect Interactions: Biosynthesis, Regulation, Ecological Functions, and Prospects for Crop Protection
Source: Plants (Basel). 2026 Aug 30;15(17):2660. doi: 10.3390/plants15172660 (PMC13567581; doi:10.3390/plants15172660)
Supplement: Supplementary file 1 [file plants-15-02660-s001.zip › plants-4484301-supplementary.pdf]

Supplementary Table S1. Study-level evidence matrix for the literature included in this review, covering cucurbitacin distribution, biosynthesis, molecular regulation, plant–herbivore interactions, and crop-protection applications.

Study	Plant species or study material	Study focus or biological target	Research question	Experimental system and methods	Main finding	Evidence tier	Main limitation
[1] Chen et al. (2005)	Multiple cucurbitacin-producing taxa	Cucurbitacin chemistry and biological activity	Context: What structural classes and biological activities of cucurbitacins had been established?	Review of phytochemical and bioactivity literature	Summarized cucurbitacin and cucurbitane-glycoside structures and their reported biological activities.	Contextual	Broad review; not designed to establish a plant biosynthetic or ecological mechanism.
[2] Thimmappa et al. (2014)	Multiple plant taxa	General plant triterpene biosynthesis	Context: What is the general biochemical framework for plant triterpene formation?	Review of plant triterpene enzymology, genomics, and pathway organization	Provided the precursor, oxidosqualene-cyclase, and tailoring-enzyme framework used to contextualize cucurbitacin biosynthesis.	Contextual	Not cucurbitacin-specific and predates several recent pathway discoveries.
[3] Cai et al. (2015)	Multiple cucurbitacin sources	Cucurbitacin phytochemistry and anticancer activity	Context: What cucurbitacin structures and anticancer activities had been summarized?	Systematic review of phytochemical and anticancer literature	Synthesized cucurbitacin chemical diversity and reported anticancer evidence.	Contextual	Pharmacological focus; limited direct relevance to plant biosynthesis or plant–herbivore ecology.
[4] Dong et al. (2021)	*Iberis amara*; selected cucurbits	Insect-deterrent pathway	Evolution: Did cucurbitacin biosynthesis evolve independently in Brassicaceae?	Comparative phylogenomics, metabolomics, heterologous enzyme assays	Distinct OSC and P450 lineages reconstruct an independently evolved *I. amara* pathway.	Validated	Native genetic and ecological validation in *I. amara* remains limited.

Study	Plant species or study material	Study focus or biological target	Research question	Experimental system and methods	Main finding	Evidence tier	Main limitation
[5] Chen et al. (2014)	*Aquilaria agallocha*	Chemical distribution / pathway candidates	Distribution: Are cucurbitacins present and are candidate biosynthetic genes detectable?	Draft genome assembly and metabolite identification	Reported cucurbitacin-related metabolites and a genomic resource for pathway searches.	Supported	Pathway candidates were not functionally validated.
[6] Mei et al. (2012)	*Aquilaria sinensis*	Fruit metabolites	Distribution: Which cucurbitacins occur in fruits?	Compound isolation and spectroscopic structure elucidation	Chemically characterized cucurbitacins from *A. sinensis* fruits.	Validated	No biosynthetic or ecological tests.
[7] Li et al. (2015)	*Aquilaria sinensis*	Leaf metabolites / indirect phytochemical context	Distribution: Does this phytochemical study directly document cucurbitacins?	Compound isolation and spectroscopy	Characterized megastigmane glycosides rather than cucurbitacins.	Contextual	Provides only indirect phytochemical context and does not directly support cucurbitacin occurrence.
[8] Yang et al. (2022)	*Aquilaria sinensis*	Flower constituents	Distribution: Which bioactive constituents occur in flowers?	Bioassay-guided isolation and spectroscopy	Identified cytotoxic constituents, including cucurbitane-related compounds reported by the study.	Validated	Bioactivity-driven sampling; no pathway or ecological validation.
[9] Ding et al. (2024)	*Aquilaria sinensis*	*Bi*-like OSC candidate	Biosynthesis: Is a cucurbitacin-associated gene cluster present in a woody plant?	Genome/transcriptome analysis, metabolite profiling, transient expression	Linked *As03G2784* and neighboring candidates to cucurbitacin production in *Aquilaria.*	Supported	Native loss-of-function and complete pathway reconstruction are lacking.
[10] Pan et al. (2012)	*Elaeocarpus chinensis*	Cucurbitane metabolites	Distribution: Which epoxycucurbitacins occur in the species?	Isolation, NMR/MS structure elucidation, bioassays	Established structures of 16,23-epoxycucurbitacin constituents.	Validated	No biosynthetic or ecological analysis.
[11] Ito et al. (2002)	*Elaeocarpus mastersii*	Cucurbitane metabolites	Distribution: Are cucurbitacins present in *E. mastersii*?	Isolation and spectroscopic identification	Chemically identified cucurbitacins in the species.	Validated	Occurrence was not linked to genes, tissues, or herbivory.

Study	Plant species or study material	Study focus or biological target	Research question	Experimental system and methods	Main finding	Evidence tier	Main limitation
[12] Cho et al. (2022)	*Elaeocarpus petiolatus*	Cucurbitane metabolites	Chemical diversity: Which unusual cucurbitacins occur?	Isolation and NMR/MS structure elucidation	Characterized pentacyclic and hexacyclic cucurbitacins.	Validated	No functional or ecological tests.
[13] Hu et al. (2023)	*Elaeocarpus dubius*	Cucurbitane heterodimers	Chemical diversity: Are cucurbitane heterodimers present?	Isolation and spectroscopic structure elucidation	Established cucurbitane-type heterodimer structures.	Validated	Biogenesis and ecological relevance remain unresolved.
[14] Meng et al. (2008)	*Elaeocarpus hainanensis*	Cucurbitane triterpenoids	Distribution: Which cucurbitane-type triterpenoids occur?	Isolation, spectroscopy, cytotoxicity assays	Chemically characterized cucurbitane-type triterpenoids.	Validated	Pharmacological focus; no plant-function evidence.
[15] Shang et al. (2014)	*Cucumis sativus*	*Bi* pathway; *Bt*/*Bl* regulators; domestication	Biosynthesis/regulation: What controls cucurbitacin C and bitterness?	Genetics, gene clustering, heterologous assays, promoter analyses, metabolite phenotyping	Validated *CsBi* and tissue-preferential bHLH regulators and linked fruit bitterness to domestication.	Validated	Most conclusions are cucumber-specific; downstream tailoring remains incomplete.
[16] Zhou et al. (2016)	Cucumber, melon, watermelon and other cucurbits	Pathway and bHLH regulators	Comparative biosynthesis: Which pathway features converge or diverge among cucurbits?	Comparative genomics, metabolomics, gene-expression and functional assays	Resolved conserved and lineage-specific features of bitterness biosynthesis and regulation.	Validated	Validation depth differs among species and pathway steps.
[17] Wang et al. (2024)	*Cucumis melo*	*CmBr*/*CmRSM1*; CPPU-induced bitterness	Regulation: How does CPPU induce bitter fruit?	Genetic analysis, promoter binding/transactivation, expression and metabolite assays	Showed that *CmBr* and upstream regulation contribute to CPPU-induced cucurbitacin B accumulation.	Validated	Field relevance and effects beyond fruit quality were not tested.

Study	Plant species or study material	Study focus or biological target	Research question	Experimental system and methods	Main finding	Evidence tier	Main limitation
[18] Luo et al. (2024)	*Cucumis melo* var. *makuwa*	*CmWRKY13*	Regulation: Does *CmWRKY13* activate cucurbitacin B biosynthesis?	Promoter assays, protein interaction and expression/metabolite analyses	Supported activation of pathway genes and interaction with *CmBt.*	Supported	Comprehensive native loss-of-function evidence is lacking.
[19] Balkema-Boomstra et al. (2003)	*Cucumis sativus*	*Tetranychus urticae*	Plant defense: Is cucurbitacin C associated with spider-mite resistance?	Contrasting genotypes, intact-plant infestation and metabolite/performance measurements	Associated cucurbitacin C with reduced mite preference and performance.	Supported	Genotype differences may include defenses other than cucurbitacin C.
[20] Yousaf et al. (2018)	Purified compound / *Aphis gossypii*	Melon aphid	Insect fitness: How does cucurbitacin B affect aphid demography?	Controlled exposure and life-table analysis	Reduced survival, longevity, fecundity and population growth.	Validated	Purified-compound exposure may not reproduce intact-plant delivery.
[21] Zhao et al. (2021)	Purified compounds / *Aphis gossypii*	Melon aphid	Biopesticide potential: Does cucurbitacin B suppress aphids?	Artificial-diet/feeding assays and physiological measurements	Confirmed dose-dependent adverse effects and altered detoxification responses.	Validated	Laboratory exposure; field efficacy and non-target effects remain uncertain.
[22] Ponsankar et al. (2020)	*Citrullus colocynthis* compound	*Spodoptera litura*; *Eisenia fetida*	Toxicity: What are target and non-target effects of cucurbitacin E?	Purified-compound dose-response, development and reproduction assays	Caused deterrence, mortality and developmental/reproductive impairment in *S. litura* and assessed earthworm effects.	Validated	Purified-compound dosing does not establish intact-plant or field performance.

Study	Plant species or study material	Study focus or biological target	Research question	Experimental system and methods	Main finding	Evidence tier	Main limitation
[23] Chambliss & Jones (1966)	Cucurbitaceae extracts/compounds	Diabroticite beetles	Behavior: Do cucurbitacins stimulate specialist beetle responses?	Feeding/attraction bioassays	Established cucurbitacins as specific behavioral cues for cucurbit-associated beetles.	Validated	Early assays did not resolve sensory range or molecular mechanism.
[24] Metcalf et al. (1980)	Cucurbitacins / cucurbit tissues	Diabroticite beetles	Behavior: Do cucurbitacins function as kairomonal feeding cues?	Comparative behavioral and feeding assays	Demonstrated strong diabroticite responses to cucurbitacins.	Validated	Long-range attraction and contact feeding stimulation were not always separated.
[25] Ferguson et al. (1985)	Purified cucurbitacin B	Five diabroticite species	Metabolism: What happens to ingested cucurbitacin B?	Feeding, chemical recovery and metabolite analysis	Documented species-specific absorption, transformation and retention.	Validated	Limited taxa and laboratory exposure; fitness consequences were not tested.
[26] Gray et al. (2009)	Maize agroecosystems; western corn rootworm	Pest adaptation and invasiveness	Context: Why is western corn rootworm a major, difficult-to-manage agricultural pest?	Review of population biology, invasion, adaptation, and management literature	Summarized the pest's adaptive capacity, invasion history, and escalating management challenge.	Contextual	Not a cucurbitacin study and does not test cucurbitacin-mediated behavior or control.
[27] Chen et al. (2022)	*Cucumis sativus* organs	Cucurbitacins/glycosides	Chemical profiling: How broadly are cucurbitacins distributed among organs?	HPLC-Q-TOF-MS with fragmentation/adduct rules	Screened 40 putative cucurbitacins and proposed organ-specific profiles.	Supported	Many annotations lacked authentic standards or NMR confirmation.
[28] Qing et al. (2022)	*Cucumis sativus* leaves	Cucurbitacin C analogues	Chemical diversity: Which bitter-leaf analogues can be structurally confirmed?	Isolation and NMR/MS structure elucidation	Characterized ten analogues, including seven previously undescribed cucurbitacins.	Validated	Restricted tissue/genotype sampling and no ecological tests.

Study	Plant species or study material	Study focus or biological target	Research question	Experimental system and methods	Main finding	Evidence tier	Main limitation
[29] Zhong et al. (2017)	*Cucumis sativus*	*UGT73AM3*	Tailoring: Which enzyme glucosylates a bitter intermediate?	Enzyme assay, heterologous expression and developmental metabolite analysis	Validated developmentally regulated glucosylation by *UGT73AM3*.	Validated	Native pathway-wide consequences and ecological effects were not evaluated.
[30] Benka et al. (2023)	*Citrullus naudinianus* tuber	Cucurbitacins/dihydrocucurbitacins	Chemical diversity: Which compounds occur in the tuber?	Mass spectrometry with structural analysis	Resolved a diverse cucurbitacin and dihydrocucurbitacin profile.	Validated	Limited to one tissue; no biosynthetic validation.
[31] Stuppner & Wagner (1989)	*Picrorhiza kurrooa*	Cucurbitacin glycosides	Distribution: Which glycosides occur outside Cucurbitaceae?	Isolation and spectroscopic structure elucidation	Characterized new cucurbitacin glycosides.	Validated	No molecular pathway or ecological evidence.
[32] Halaweish (1993)	*Cucurbita texana*	Isocucurbitacins	Chemical diversity: What is the role of isocucurbitacins in the profile?	Compound isolation/analysis and chemical comparison	Provided chemical evidence for isocucurbitacins in *C. texana*.	Validated	Biological function and biosynthetic origin were not resolved.
[33] Ul Haq et al. (2019)	Ten Cucurbitaceae species	Cucurbitacin profiles	Distribution: How do profiles vary among species and genera?	Comparative LC-MS/MS profiling and quantitation	Found species-specific combinations of cucurbitacins B, E, I and E-glucoside.	Supported	Taxon/tissue coverage and analytical standards were limited.
[34] Min et al. (2023)	69 *Cucumis sativus* inbred lines	Fruit cucurbitacins and volatiles	Domestication/variation: How variable are fruit chemical traits?	Targeted metabolite, volatile and morphological profiling	Revealed strong genotypic variation and patterns consistent with independent domestication.	Supported	Association study; causal loci and herbivore outcomes were not tested.
[35] Qing et al. (2014)	*Cucumis sativus*	23,24-Dihydrocucurbitacin C	Metabolism: Is the dihydro derivative a downstream metabolite?	Isolation, structure elucidation and comparative occurrence	Identified the derivative and proposed its position after cucurbitacin C.	Supported	The conversion enzyme and in vivo flux were not demonstrated.

Study	Plant species or study material	Study focus or biological target	Research question	Experimental system and methods	Main finding	Evidence tier	Main limitation
[36] Fan et al. (2024)	Bitter cucumber fruit	Cucurbitacin C	Food chemistry: How does concentration vary by tissue/development and processing?	Targeted quantitation, fermentation and acidification experiments	Quantified fruit heterogeneity and reduction during processing.	Validated	Single-cultivar/processing context; not a biosynthetic or ecological test.
[37] Robinson et al. (1976)	Cucurbitaceae, including *Cucurbita*	Bitterness loci	Genetics: What classical inheritance patterns underlie cucurbit traits?	Compilation of genetic segregation evidence	Reported simple Mendelian control of fruit bitterness in *Cucurbita*.	Supported	Classical locus-level evidence without molecular gene identification.
[38] Zhao et al. (2022)	*Luffa acutangula*	*LaBi*/*LaAREB1* and pathway candidates	Biosynthesis/regulation: What distinguishes bitter and non-bitter fruit?	Integrated metabolome/transcriptome analysis plus promoter assays	Associated multiple cucurbitacins and pathway genes with bitterness and supported *LaAREB1* activation of *LaBi*.	Supported	*LaBi* product and native regulator function were not fully validated.
[39] Ma et al. (2023)	*Cucumis sativus*	*CsMATE1* transporter	Transport: How is cucurbitacin C sequestered in leaf cells?	Genetics, localization, transport and metabolite assays	Validated *CsMATE1*-mediated vacuolar sequestration in mesophyll cells.	Validated	Generality across tissues, compounds and species remains unknown.
[40] Agrawal et al. (1999)	*Cucumis sativus*	*Tetranychus urticae*	Induction: Does herbivory increase cucurbitacin C locally/systemically?	Intact-plant herbivory, genotype comparison and metabolite quantitation	Demonstrated local and systemic induction in bitter cucumber.	Validated	Mechanistic signaling sequence and broad taxonomic generality were not resolved.

Study	Plant species or study material	Study focus or biological target	Research question	Experimental system and methods	Main finding	Evidence tier	Main limitation
[41] Tallamy (1985)	*Cucurbita pepo* (zucchini)	*Epilachna borealis* / leaf damage	Induction/behavior: Does leaf damage increase cucurbitacins and shape trenching?	Leaf-damage, chemical and feeding-behavior experiments	Reported damage-induced increases in cucurbitacins B and D and linked specialist behavior to induced defenses.	Validated	Damage and insect-specific signaling were not fully separated.
[42] McCloud et al. (1995)	*Cucurbita* spp.	*Epilachna borealis*	Induction: Does trenching avoid cucurbitacin accumulation or mucilaginous sap?	Feeding-trench comparisons and metabolite measurements	Detected no cucurbitacin accumulation inside or outside trenches and supported an alternative sap-related explanation.	Validated	Specific plant-insect context; does not exclude induction in other systems.
[43] Bruno et al. (2023)	*Cucumis sativus*	*Diabrotica balteata* and natural enemies	Sequestration/tritrophic effects: Does acquired cucurbitacin protect larvae?	Plant feeding, chemical analysis, predator/pathogen/nematode assays	Found sequestration but little consistent protection against biological-control agents.	Validated	Protection may vary with enemy, dose, plant genotype and field context.
[44] Shibuya et al. (2004)	*Cucurbita pepo*	*CPQ* cucurbitadienol synthase	Scaffold formation: Is the committed OSC distinct from cycloartenol synthase?	Heterologous enzyme expression and product identification	Validated *CPQ* as a distinct cucurbitadienol synthase.	Validated	Downstream native pathway and regulation were not addressed.
[45] Dai et al. (2015)	*Siraitia grosvenorii*	OSC and glycosyltransferase	Cucurbitane biosynthesis: Which enzymes form and glycosylate mogroside intermediates?	Heterologous expression and enzyme assays	Functionally characterized a scaffold-forming OSC and glycosyltransferase.	Validated	Mogroside pathway; extrapolation to cucurbitacins is limited.

Study	Plant species or study material	Study focus or biological target	Research question	Experimental system and methods	Main finding	Evidence tier	Main limitation
[46] Takase et al. (2019)	Selected bitter-plant systems	Cytochrome P450 enzymes	Oxidative tailoring: How does allylic hydroxylation diversify bitter triterpenoids?	Heterologous enzyme assays and product structural analysis	Demonstrated P450-catalyzed transformations generating diverse bitter compounds.	Validated	Not all products or enzymes are cucurbitacin-specific.
[47] Lertphadungkit et al. (2021)	*Trichosanthes cucumerina*	Triterpenoid pathway candidates	Candidate discovery: Which transcripts correlate with triterpenoid biosynthesis?	De novo transcriptomics and expression analysis	Identified candidate OSC, P450 and related genes.	Candidate	No direct enzyme products, genetics or cucurbitacin flux measurements.
[48] Zhang et al. (2022)	*Hemsleya macrosperma*	*HmSE1* and pathway candidates	Biosynthesis: Which genes associate with cucurbitacin IIa accumulation?	Comparative transcriptomics, expression-metabolite association and *HmSE1* characterization	Linked precursor-pathway expression to cucurbitacin IIa and supported *HmSE1* activity.	Supported	Most downstream candidates were not functionally tested.
[49] Li et al. (2023)	*Hemsleya chinensis*	*HcOSC6*	Enzyme mechanism: Which residues control cucurbitadienol synthase activity?	Heterologous expression and site-directed mutagenesis	Identified key active-site residues affecting *HcOSC6* catalysis.	Validated	Heterologous mechanism does not alone define native flux control.
[50] Chen et al. (2024)	*Hemsleya chinensis*; yeast; *Nicotiana benthamiana*	*HcSE1/2*, *HcOSC6*, *HcCYP81Q59*, *HcAT1*	Pathway reconstruction: Which steps produce cucurbitacin IIa intermediates?	Engineered yeast/tobacco modules with product identification	Reconstructed key precursor, scaffold, oxidation and acetylation steps and improved intermediate production.	Validated	Native regulation, transport and complete endpoint pathway remain incomplete.

Study	Plant species or study material	Study focus or biological target	Research question	Experimental system and methods	Main finding	Evidence tier	Main limitation
[51] Wang et al. (2024)	*Hemsleya chinensis*	*HcOSC6*	Native validation: Does *HcOSC6* control cucurbitadienol abundance in planta?	Regeneration, overexpression and CRISPR/Cas9 genome editing	Overexpression increased and editing reduced cucurbitadienol, validating native function.	Validated	One gene/plant system; ecological consequences were not tested.
[52] Almeida et al. (2022)	*Cucurbita pepo* hairy roots	Cucurbitacin pathway engineering	Metabolic engineering: Can pathway output be altered in transformed roots?	*Rhizobium*-mediated hairy roots, gene expression and metabolite profiling	Demonstrated engineered changes in cucurbitacin-related metabolism.	Supported	Hairy-root transformation itself perturbs metabolism and is not an intact crop.
[53] Almeida et al. (2023)	Cucurbitaceae hairy roots	Transformation-system effects	Methodological validation: How strongly does hairy-root transformation alter host metabolism?	Comparative transcriptome and metabolome profiling	Showed extensive transformation-associated changes that can confound pathway interpretation.	Supported	System-focused study; findings do not directly validate cucurbitacin genes.
[54] Li et al. (2017)	Engineered yeast	Cucurbitadienol synthesis	Heterologous production: How can cucurbitadienol yield be increased?	Yeast pathway construction and product quantitation	Demonstrated heterologous cucurbitadienol production and optimization.	Validated	Microbial flux does not establish plant regulation or agronomic value.
[55] Wang et al. (2022)	*Saccharomyces cerevisiae*	Mogrol pathway	Heterologous production: Which engineering steps improve mogrol synthesis?	De novo yeast pathway construction and optimization	Improved production of a related cucurbitane-type triterpenoid.	Validated	Targets mogrol, not cucurbitacin; crop extrapolation is indirect.
[56] Xu et al. (2022)	Seven cucurbit crops	bHLH family / *Brp* candidate	Comparative regulation: How did bitterness-associated bHLHs diversify?	Comparative genomics, phylogeny and expression association	Identified conserved and duplicated bHLH clusters including a root-associated candidate.	Candidate	Candidate *Brp* function lacks direct native genetic and promoter validation.

Study	Plant species or study material	Study focus or biological target	Research question	Experimental system and methods	Main finding	Evidence tier	Main limitation
[57] Wang et al. (2026)	*Cucumis melo*	*CmBt* and *CmBr*	Combinatorial regulation: Do the two bHLHs act synergistically?	CRISPR/Cas9 single/double mutants, protein interaction, promoter and metabolite assays	Demonstrated overlapping, tissue-biased and synergistic control of cucurbitacin B, including CPPU-induced bitterness.	Validated	Field performance and herbivore-community effects were not evaluated.
[58] Hua et al. (2019)	*Cucumis melo* fruit	Cucurbitacin B and pathway transcripts	Development: How do bitterness, metabolite and gene expression change during ripening?	Developmental time series with metabolite and expression measurements	Found high early levels in bitter genotypes followed by decline during maturation.	Supported	Correlative time series; causal regulators and turnover mechanisms were not tested.
[59] Tian et al. (2025)	*Cucumis melo* germplasm	Developmental network / ABC candidates	Development/transport: What explains contrasting cucurbitacin B decline patterns?	Integrated spatiotemporal transcriptomics and metabolomics	Associated hormone, senescence, terpenoid and ABC-transporter expression with cucurbitacin B dynamics.	Candidate	Correlations do not demonstrate transport, turnover or causal regulation.
[60] Kuo et al. (2015)	*Aquilaria agallocha* tissues	Light-responsive secondary metabolism	Environment: Do red/far-red light conditions alter cucurbitacin-related metabolism?	Light treatments with metabolite, transcript, small-RNA and methylation profiling	Associated light conditions with secondary-metabolic and regulatory changes.	Supported	Direct pathway regulators and causal links to individual cucurbitacins were not established.
[61] Jaccard et al. (2023)	Wild and domesticated *Cucurbita argyrosperma*	*Diabrotica balteata*; *Acalymma* spp.	Domestication: How has crop selection altered defense and herbivore interactions?	Common-garden plant chemistry/expression plus root preference and performance assays	Domestication reduced cucurbitacins; specialist taxa differed in preference and performance responses.	Validated	Limited genotypes and controlled conditions; other correlated traits may contribute.

Study	Plant species or study material	Study focus or biological target	Research question	Experimental system and methods	Main finding	Evidence tier	Main limitation
[62] Brzozowski et al. (2020)	*Cucurbita pepo* domestication lineages	*Acalymma vittatum*	Domestication genetics: How do independent events alter cucurbitacins and specialist preference?	Metabolite phenotyping, genetic mapping and beetle preference assays	Linked divergent cucurbitacin profiles and a major locus to differences in specialist preference.	Supported	Candidate genes within the locus were not functionally validated.
[63] Ye et al. (2024)	Wild and cultivated *Cucurbita argyrosperma*	Above- and belowground herbivores	Induced defense: Are responses cultivar-, organ- and herbivore-specific?	Controlled herbivory with gene-expression, trichome and chemical/plant-trait measurements	Found cultivar- and organ-specific responses; gene/trait changes did not uniformly indicate cucurbitacin induction.	Supported	Causal links among expression, chemistry and herbivore performance remain incomplete.
[64] Milano et al. (2015)	*Cucumis sativus*	Aboveground insects; *Acalymma vittatum* larvae	Systemic response: Does shoot herbivory alter later root preference via cucurbitacin C?	Sequential intact-plant herbivory, root-choice, biomass and metabolite assays	Aboveground herbivory reduced root preference without increasing leaf or root cucurbitacin C.	Validated	Mechanism was not resolved and root biomass covaried with preference.
[65] Tallamy et al. (1997)	Cucurbitacin-treated substrates	Multiple insect species	Behavior: How broadly do cucurbitacins deter feeding and oviposition?	Multi-species choice and oviposition assays	Demonstrated deterrence in several sensitive taxa but heterogeneous responses among guilds.	Validated	Artificial substrates and limited compounds reduce intact-plant generality.

Study	Plant species or study material	Study focus or biological target	Research question	Experimental system and methods	Main finding	Evidence tier	Main limitation
[66] Lang et al. (2013)	Purified/modified cucurbitacins	Generalist and specialist insects	Structure-activity: Do chemical modifications change antifeedant responses?	Comparative dose-response feeding assays	Small structural changes produced species-specific deterrent or stimulatory effects.	Validated	Limited species/derivatives and laboratory exposure.
[67] Metcalf et al. (1982)	*Cucurbita* spp.	Diabroticite beetles	Behavior-chemistry: Does tissue cucurbitacin content predict feeding?	Plant chemical quantitation and comparative feeding assays	Found positive relationships between cucurbitacin content and diabroticite feeding/arrestment.	Supported	Correlated plant traits and contact responses limit causal separation.
[68] Ferguson et al. (1983)	19 species / 46 cucurbit cultivars	Diabroticite beetles	Comparative behavior: How do cotyledon profiles influence feeding?	Cotyledon chemical analysis and multi-taxon feeding assays	Showed strong effects of cucurbitacin identity and concentration on beetle feeding.	Supported	Cotyledon assays may not predict adult host use or field damage.
[69] Chyb et al. (1995)	Cucurbitacin B stimulus	*Diabrotica virgifera virgifera* adults	Sensory mechanism: Which sensilla mediate cucurbitacin taste?	Electrophysiology and bilateral sensillum removal with feeding assays	Identified maxillary galeal sensilla required for cucurbitacin-stimulated feeding.	Validated	One life stage/species; does not establish long-range attraction.
[70] Andersen et al. (1988)	Purified cucurbitacin D	*D. virgifera virgifera*; *D. undecimpunctata howardi*	Metabolism/sequestration: How is ingested cucurbitacin transformed?	Feeding, transfer to clean diet and chemical metabolite analysis	Documented glucosylation, hydrogenation, desaturation, acetylation and persistent retention.	Validated	Laboratory dosing; adaptive fitness value was not tested.

Study	Plant species or study material	Study focus or biological target	Research question	Experimental system and methods	Main finding	Evidence tier	Main limitation
[71] Jaccard et al. (2022)	Wild/domesticated *Cucurbita argyrosperma* roots	*D. balteata* and predatory rove beetle	Tritrophic effects: Does domestication-mediated chemistry alter predator-prey outcomes?	Belowground plant-herbivore-predator experiment with chemistry and attack measurements	High-cucurbitacin feeding did not improve larval protection and may impose processing costs.	Validated	One predator and controlled system; field multitrophic generality is unknown.
[72] Wallingford et al. (2025)	Watermelon-derived cues / bait system	Striped and spotted cucumber beetles	Field control: Can multiple cues improve attract-and-kill performance?	Multicomponent behavioral lures and field trapping/feeding tests	Combining pheromone, host cues and cucurbitacin E glycoside increased responses, with strong design/placement effects.	Validated	Location- and design-dependent performance; non-target effects require evaluation.
[73] Pedersen & Godfrey (2011)	Melon fields	Cucumber beetles	Field control: Does a cucurbitacin stimulant improve foliar insecticides?	Replicated field trials with stimulant plus spinosad/carbaryl	The stimulant did not reliably improve beetle control.	Validated	Formulation, pest density and environmental context may limit transferability.
[74] Avila-Sakar & Stephenson (2006)	Wild *Cucurbita pepo* subsp. *texana*	Simulated foliar damage	Tolerance: How do damage intensity and spatial pattern affect plant fitness?	Whole-plant damage experiment with growth and reproduction measurements	Showed that tolerance depends on damage pattern and intensity.	Validated	Mechanical damage is not a cucurbitacin-specific or insect-specific mechanism.
[75] Thompson et al. (2025)	Six cucurbit taxa	Above- and belowground insect herbivores	Resistance/tolerance: How do defense responses vary by organ and evolutionary history?	Comparative herbivory experiments with plant performance and defense traits	Found distinct shoot/root defenses shaped by eco-evolutionary context and no uniform domestication trend.	Supported	Broad trait analysis; direct cucurbitacin causality is limited.

Study	Plant species or study material	Study focus or biological target	Research question	Experimental system and methods	Main finding	Evidence tier	Main limitation
[76] Vaughn et al. (2022)	*Cucumis melo* accessions	Graph pangenome / fungal resistance	Genomic resources: Can structural variation improve trait mapping?	Graph-pangenome construction and association mapping	Increased genotyping density and resolved structural effects on resistance loci.	Supported	Not cucurbitacin- or insect-specific; functional validation is still required.
[77] Zhang et al. (2024)	All seven *Citrullus* species	Watermelon super-pangenome	Genomic resources: What variation is captured across *Citrullus*?	Telomere-to-telomere assemblies, super-pangenome and trait analyses	Revealed extensive structural variation relevant to domestication and breeding.	Supported	Genomic association does not validate cucurbitacin genes or insect resistance.
[78] Nie et al. (2025)	*Cucumis sativus*	*NPF1*, parthenocarpy and *Bt* regulation	Pleiotropy: How is fruit development linked to bitterness?	Genetic, molecular and metabolite analyses	Showed that *NPF1* affects parthenocarpy and suppresses *Bt*/cucurbitacin C biosynthesis.	Validated	Crop-wide ecological consequences and field stability were not tested.
[79] Uranga et al. (2024)	Multiple crop systems	CRISPR–Cas crop bioengineering	Context: Which genome-editing systems could support functional validation and crop engineering?	Review of CRISPR–Cas systems, delivery approaches, and crop applications	Outlined genome-editing platforms and application constraints relevant to future pathway-gene validation.	Contextual	Not cucurbitacin-specific; does not validate a cucurbitacin target or agronomic outcome.
[80] Meinke et al. (2021)	Maize agroecosystems; *Diabrotica virgifera virgifera*	Insecticide use and resistance management	Context: How has western corn rootworm management generated and responded to field-evolved resistance?	Review of insecticide history, field resistance, and resistance mechanisms	Documented repeated resistance evolution and the limitations of insecticide-centered management.	Contextual	Maize-pest management review; no direct test of cucurbitacin-based management.

Study	Plant species or study material	Study focus or biological target	Research question	Experimental system and methods	Main finding	Evidence tier	Main limitation
[81] Zhang et al. (2025)	Multiple crops and insect pests	Insect-resistant crops expressing double-stranded RNA	Context: What is the development status and future potential of plant-mediated RNA interference?	Review of dsRNA targets, crop expression strategies, efficacy, and translation	Summarized the development and prospects of insect-resistant crops that express double-stranded RNAs.	Contextual	Not cucurbitacin-specific; efficacy, durability, and regulatory translation remain system-dependent.
[82] Luo et al. (2024)	RNAi-based biopesticide systems	Environmental and biosafety risk assessment	Context: Which risks should be assessed before RNAi-based biopesticides are deployed?	Review of exposure, persistence, off-target, and non-target risk frameworks	Outlined key hazard and exposure considerations for RNAi-based crop-protection products.	Contextual	Methodological risk framework; no cucurbitacin-specific efficacy or ecological test.
[83] Demurtas et al. (2023)	Multiple plant taxa	Terpenoid transport	Context: What is known about cellular and long-distance transport of plant terpenoids?	Review of transporter classes, trafficking routes, and unresolved mechanisms	Provided a general framework for interpreting candidate transport processes in specialized metabolism.	Contextual	Not cucurbitacin-specific and does not validate the transporter candidates discussed in this review.
[84] Zhang et al. (2024)	*Camellia sinensis* and associated pests/pathogens	Specialized-metabolite-mediated defense	Context: How can specialized metabolites contribute to resistance against insects and pathogens?	Review of tea metabolites and plant–pest/pathogen interactions	Summarized multiple metabolite classes and defense functions in a crop protection context.	Contextual	Different plant and chemical systems; supports only general defense concepts.

Study	Plant species or study material	Study focus or biological target	Research question	Experimental system and methods	Main finding	Evidence tier	Main limitation
[85] Yin et al. (2024)	Plant tissues and metabolomics workflows	Mass spectrometry imaging	Context: Which imaging approaches can resolve the spatial distribution of plant metabolites?	Methods review of mass spectrometry imaging platforms and applications	Summarized spatial-metabolomics capabilities relevant to mapping specialized metabolites in tissues.	Contextual	Method-focused; provides no direct cucurbitacin spatial map or functional validation.
[86] Schreiber et al. (2024)	Multiple crop pangenomes	Pangenomics for crop improvement	Context: How can pangenomes reveal diversity relevant to breeding and evolutionary analysis?	Review of pangenome construction, structural variation, diversity, and crop applications	Established the conceptual basis for using pangenomes to discover structural variation and breeding targets.	Contextual	Not cucurbitacin- or cucurbit-specific; genomic associations require downstream functional validation.
[87] Zhao et al. (2026)	*Cucumis sativus* breeding panel	Graph pangenome	Genomic resources: How has structural variation changed during cucumber breeding?	Graph-pangenome construction, population analysis and trait mapping	Mapped structural-variation dynamics and expanded candidate discovery during breeding.	Supported	Candidate associations require metabolite, gene-function and herbivore validation.

Evidence tiers were assigned relative to the research question specified for each study. Validated—direct experimental evidence addressing the stated research question through chemical structure elucidation, biochemical or product-based assays, genetic manipulation, transport assays, or causal ecological tests; Supported—convergent but incomplete evidence from intact-plant associations, transient assays, promoter analyses, multi-omics analyses, or pathway reconstruction without full native causal validation; Candidate—inference based primarily on genomic, transcriptomic, co-expression, phylogenetic, or computational evidence without direct functional validation; Contextual—background, methodological, economic, or general conceptual evidence not used to establish a cucurbitacin-specific mechanism.
